# Immunosuppressants in Liver Transplant Recipients With Coronavirus Disease 2019: Capability or Catastrophe?—A Systematic Review and Meta-Analysis

**DOI:** 10.3389/fmed.2021.756922

**Published:** 2021-11-11

**Authors:** Dipesh Kumar Yadav, Vishnu Prasad Adhikari, Qi Ling, Tingbo Liang

**Affiliations:** ^1^Department of Hepatobiliary Surgery & Pancreatic Surgery, The First Affiliated Hospital, Zhejiang University, Hangzhou, China; ^2^Zhejiang Provincial Key Laboratory of Pancreatic Disease, Hangzhou, China; ^3^Zhejiang Provincial Innovation Center for the Study of Pancreatic Diseases, Hangzhou, China; ^4^Zhejiang Provincial Clinical Research Center for the Study of Hepatobiliary & Pancreatic Diseases, Hangzhou, China; ^5^Cancer Center, Zhejiang University, Hangzhou, China

**Keywords:** COVID-19, SARS-CoV-2, immunosuppression, liver transplant, coronavirus

## Abstract

**Background:** The probable impact of a maintenance immunosuppressant (IS) on liver transplant (LT) recipients with coronavirus disease 2019 (COVID-19) remains unexplored. Our specific aim was to approximate the prognosis of LT recipients with COVID-19 on the standard maintenance IS.

**Method:** We searched separate databases for the qualified studies in between December 2019 and June 25, 2021. Ultimately, a meta-analysis was carried out using a fixed-effect or random-effect model based on the heterogeneity.

**Results:** In a total of eight studies and 509 LT recipients with COVID-19, the pooled rates of severity and mortality during all the combined immunosuppressive therapies were 22.4 and 19.5%, respectively. Our study sufficiently showed that an immunosuppressive therapy in LT recipients with COVID-19 was significantly associated with a non-severe COVID-19 [odds ratio (OR): 11.49, 95% CI: 4.17–31.65; *p* < 0.001] and the survival of the patients (OR: 17.64, 95% CI: 12.85–24.22; *p* < 0.001). Moreover, mammalian target of rapamycin inhibitor (mTORi) typically had the lowest rate of severity and mortality compared to other ISs such as calcineurin inhibitors (CNIs), steroids, and antimetabolites, i.e., severity (13.5 vs. 21.1, 24.7, and 26.3%) and mortality (8.3 vs. 15, 17.2, and 12.1%), respectively. Contrary to the general opinions, our meta-analysis showed comorbidities such as diabetes, hypertension, cardiopulmonary disorders, chronic kidney disease (CKD), age >60, the duration of LT to the diagnosis of COVID-19, primary disease for LT, and obesity were not significantly associated with the severity and mortality in LT recipients with COVID-19 under an immunosuppressive therapy. However, our pooled analysis found that LT recipients with COVID-19 and without comorbidities have a less severe disease and low mortality rate compared to those with both COVID-19 and comorbidities.

**Conclusions:** In conclusion, LT recipients with COVID-19 undergoing immunosuppressive therapies are not significantly associated with the severity and mortality. Therefore, taking the risk of organ rejection into a key consideration, a complete withdrawal of the IS may not be wise. However, mycophenolate mofetil (MMF) might be discontinued or replaced from an immunosuppressive regimen with the CNIs- or mTORis-based immunosuppressive therapy in some selected LT recipients with COVID-19, depending upon the severity of the disease.

## Introduction

The coronavirus disease 2019 (COVID-19), which is typically caused by the severe acute respiratory syndrome coronavirus 2 (SARS-CoV-2), is extremely contagious and often entails significant mortality (1). From a unique perspective of the antiviral immunity, both a healthy immune system and an ample viral exposure are naturally required for a robust immune response (2). Because the immune responses in an immunosuppressed patient could be suboptimal, a specific concern typically exists regarding the potential vulnerability of liver transplant (LT) recipients to a severe COVID-19, who conventionally belong to an immunosuppressed population due to an immunosuppressant (IS) drug routinely purposed to prevent the organ rejection.

Broadly, a maintenance IS traditionally prescribed for LT recipients are calcineurin inhibitors (CNIs), antiproliferative/antimetabolites [mycophenolate mofetil (MMF), mycophenolic acid (MPA), and azathioprine (AZA)], corticosteroids, and mammalian target of rapamycin inhibitors (mTORis). These ISs traditionally act on a specific target within the three signals of T-cell activation and proliferation (3). The precise mechanism of action of these ISs customarily includes the inhibition of the production and release of cytokines from activated T-cells, downregulation/inhibition of the receptors on the T-cell, and inhibition of T-cell proliferation and T-cell depletion. Notably, there is predominantly a high prevalence of hypogammaglobulinemia after LT due to an immunosuppression, frequently associated with an increased risk of numerous viral infections such as Epstein–Barr virus, cytomegalovirus, and respiratory syncytial virus (4, 5). Moreover, due to an impaired immune defense from both the underlying disease and an immunosuppressive treatment, LT recipients substantially have a high prevalence of various comorbidities and active malignancy (6). Thus, they are invariably at a considerable risk of more severe infection and mortality due to COVID-19 compared with their immunocompetent counterpart. Additionally, it is also hypothesized that the IS in LT recipients can invariably lead to an increase in viral load and a delayed recovery from COVID-19 (7, 8). Therefore, currently, there is an ongoing debate on whether to continue, discontinue, or modify the standard doses of the maintenance IS in these patients (6, 9, 10). However, there is likewise a genuine concern that a marked reduction in IS doses or their discontinuation may cause a graft rejection and an immune reconstitution syndrome (IRS) causing a worsening of paradoxical disease (11). Nevertheless, recent studies suggest that LT recipients may not be at an increased risk of severe COVID-19 (12), and ISs may execute a protective role through modulating an immune host response to COVID-19 (13).

In the apparent absence of compelling evidence, there is a strong dependence on an experience based on the previous similar epidemics such as severe acute respiratory syndrome coronavirus (SARS) and middle east respiratory syndrome (MERS), and from a consensus based on an expert opinion. Therefore, we aimed to perform a meta-analysis to properly estimate the prognosis of LT recipients with COVID-19 on the standard maintenance IS.

At present, no meta-analysis has succinctly summarized the available findings of LT recipients with COVID-19 on the maintenance IS in depth. For this apparent reason, we sincerely believe that our meta-analysis is the first to carefully scrutinize the possible outcome of an immunosuppressive therapy in LT recipients with COVID-19 and approximate the possible prognosis of LT recipients with COVID-19 thoroughly under the standard maintenance immunosuppressive therapy.

## Methods

### Study Search Strategy

We properly conducted this systematic review and meta-analysis according to the Preferred Reporting Items for Systematic Reviews and Meta-Analyses (PRISMA) Statement. The databases such as PubMed, EMBASE, Scopus, Cochrane Library databases, and Web of Sciences were carefully searched for the relevant papers by the two authors (DKY and VPA) independently with the priorly settled convention. The last search was performed on June 25, 2021. An extensive search for the published articles in these databases was carried out with the proper use of the following Medical Subject Headings (MeSH) and non-MeSH terms: “COVID-19,” “Novel coronavirus 2019,” “Coronavirus Disease 2019,” “2019-nCoV,” “SARS-CoV-2,” “severe acute respiratory syndrome coronavirus 2,” “immunosuppression,” “immunosuppressive therapy,” “transplantation,” and “liver transplant.” Our extensive search was typically limited to the published articles in English only. Additionally, the reference lists of the reviewed articles were also screened to properly identify further relevant studies.

### Eligibility Criteria

Typically considering the outcome goals and ensuring the quality of this meta-analysis, we considered only the fully published studies (both retrospective and prospective studies) and rigorously excluded the publications such as review articles, editorials, case reports, conferences, letters, studies with the duplicate data from the same institution, studies with a multiorgan transplant, studies without a maintenance immunosuppressive therapy for LT recipients with COVID-19, and studies without a human subject. The inclusion criteria remain as follows: (a) study population: case series with four or more cases of adult LT recipients with COVID-19 and (b) comparative studies: the studies that properly compared the severe and non-severe or survivor or non-survivor and died or discharged cases of LT recipients with COVID-19.

### Data Extractions and Outcomes

All the duplicate studies were carefully excluded by using the EndNote X 8.0 software. The two investigators (DKY and VPA) who performed the literature search also independently extracted the necessary data from the included studies. Moreover, in the event of insufficient data, investigators were approached to collect more relevant results. Disagreements were adequately resolved with a third investigator. Microsoft Excel was used to accurately record all the obtainable information such as author, the year of study, institution, country, study design and characteristics, sample size, patient demographics, comorbidities, interval after the transplantation, the number of participants in the severe, non-severe, survivor, and non-survivor groups, the duration of the symptoms before and after the diagnosis, baseline IS, change in an IS after the diagnosis of COVID-19, treatment administered for COVID-19, the duration of hospital stay, and follow-up time.

The primary objective was to thoroughly evaluate the severity and mortality in LT recipients with COVID-19 undergoing different standard maintenance immunosuppressive therapies with CNIs, antiproliferatives/antimetabolites (MMF, MPA, and AZA), corticosteroids, and mTORi after the confirmed diagnosis of COVID-19. For the specific purpose of this meta-analysis, we only analyzed the standard maintenance immunosuppressive therapy that was given after the diagnosis of COVID-19, and baseline IS before the diagnosis of COVID-19 was not taken into consideration for an analysis. In case of the modification of an immunosuppressive regimen after the diagnosis of COVID-19 was not clarified, we reasonably assumed that the baseline immunosuppressive therapy was however continued in those patients after the diagnosis of COVID-19. Additionally, if, in a case, an immunosuppressive therapy was totally discontinued in any patient, we did not consider these patients in our analysis. Otherwise, as most of the patients were on an immunosuppressive regimen (dual or triple IS drugs), and less often, on a single IS drug. Therefore, we carefully extracted the data of an individual IS drug irrespective of the regimen to synthesize an analysis as it was difficult to categorize the patients based on the regimen. Considerably, a comparative analysis was also carried out for an overall immunosuppressive therapy irrespective of the usage of a single, double, or triple IS agent as a maintenance immunosuppressive therapy after the confirmed diagnosis of COVID-19.

### Definitions

In most of the studies, the diagnosis of COVID-19 and its classification were usually done according to the WHO guidance (14).

The severity of COVID-19 was often defined according to the studies, which were primarily based on the chest radiography, clinical examination, and presenting symptoms at the time of diagnosis (6, 10, 15). Non-severe COVID-19 was typically considered if LT recipients with COVID-19 were managed in an outpatient clinic or the patients admitted in a hospital, but did not undergo invasive procedures or intensive treatment. Similarly, severe COVID-19 was reasonably considered if LT recipients with COVID-19 underwent intensive treatment in a hospital, the need for admission in an intensive care unit (ICU), the progression of the disease, patients with the pulse oxygen saturation (SpO2) ≤ 90%, and a patient with an acute respiratory distress syndrome (ARDS) (6, 10, 12, 16–18). A clinical outcome of the disease was precisely defined as a survivor or non-survivor and died or discharged (6, 10, 12, 16–18).

### Risk of Bias Assessment

The quality of the included studies was rigorously evaluated using the Newcastle–Ottawa scale (NOS) (19). The scale typically comprises three assessment factors: (1) the assessment of a selection of the study groups; (2) comparability of the two groups; and (3) the outcome assessment. The NOS ranges from 0 to 9. Studies with scores of seven points and above were of high quality, those with 4–6 points were considered to be of moderate quality, and those with <4 points were considered to be of lower quality (Supplementary Table 1).

### Statistical Analyses of Data

All the data collected from the included studies were double-checked. A pooled meta-analysis was carried out using an OpenMeta Analyst, and all other meta-analyses were carried out using the RevMan Version 5.3 (Review Manager, Copenhagen: The Nordic Cochrane Center, The Cochrane Collaboration, 2014). The outcomes are presented as the pooled odds ratios (ORs) with 95% CIs. Fixed-effect or random-effect models were used to estimate a summary according to the evaluation of the heterogeneity. The *Z*-test was used to evaluate an overall effect, and the heterogeneity was assessed by using Cochran's χ^2^-test. The *I*^2^ statistic was used to evaluate the heterogeneity, which was considered as low, moderate, or high with *I*^2^ esteems >25, >50, and >75%, respectively. Two-sided *p* < 0.05 was considered as statistically significant.

## Results

### Study Search and Included Studies

**Figure 1:**
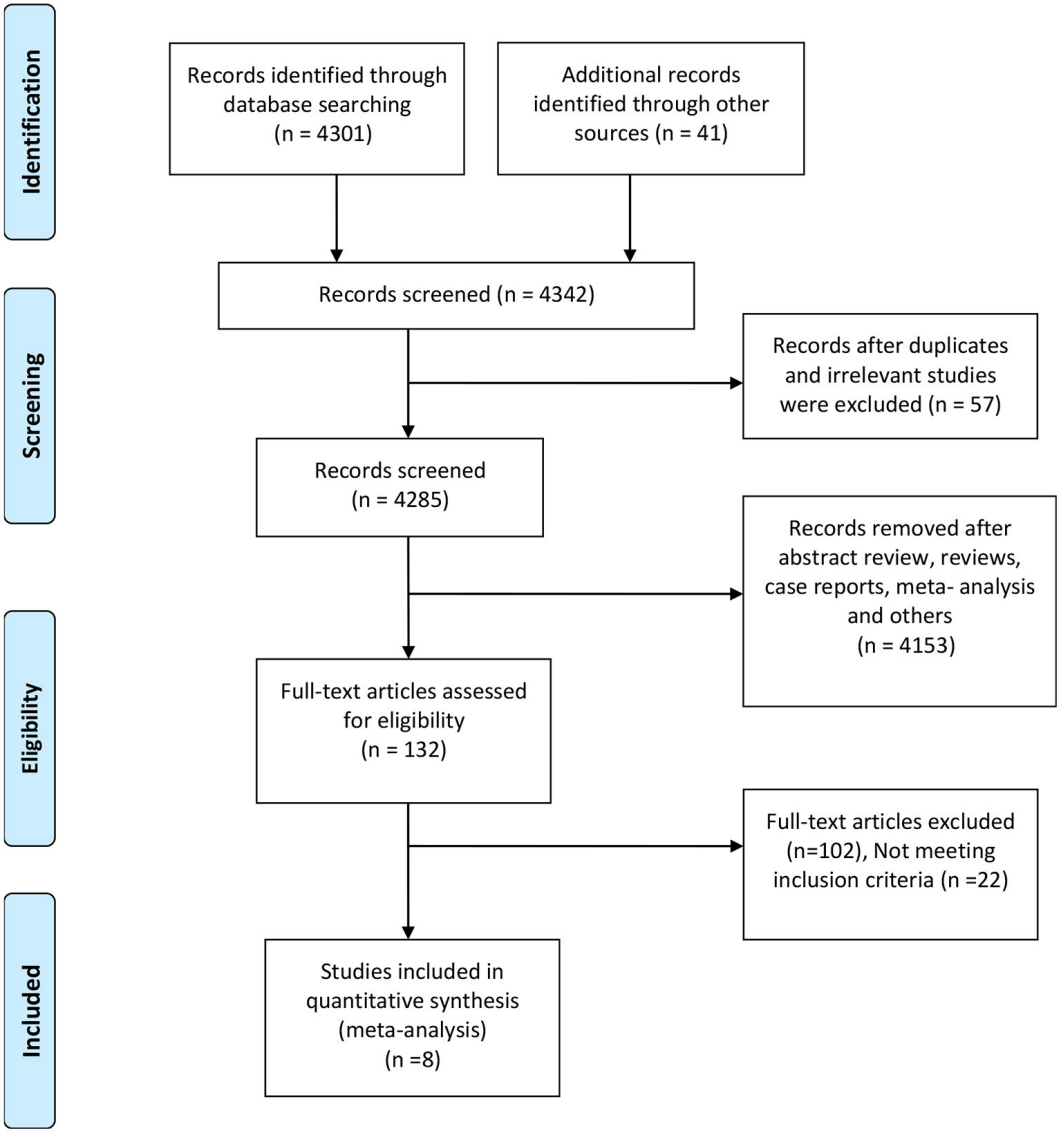
Preferred reporting items for the systematic review and meta-analysis study flow diagram for the literature search.

The database scan recognized 4,342 references for assessment, and 132 full-text articles were assessed for their eligibility. Furthermore, 124 articles were excluded for not meeting the inclusion criteria or with insufficient data. The remaining eight retrospective studies (6, 9, 10, 12, 15–18) with a total of 509 patients were eligible according to the inclusion criteria (Figure 1). The main characteristics of the included studies in our meta-analysis and the NOS score of an eligible study are presented in Table 1. The eight included studies scored between 7 and 9. According to the NOS assessment, all the included studies were considered to have a low risk of bias in selection. Publication bias was ruled out by a funnel plot (Supplementary Figure 1).

**Table 1:** Characteristics of the included studies.

**Study ID**	**References**	**Country and Institute**	**Study period**	**Study type**	**Age (Years)**	**Sex**		**Total Pts**.	**Arms**	**No. of patients**	**Comorbidities**	**Maintenance IS**	**NOS**
						**M**	**F**						
1	Verma et al. (16)	UK, Kings College Hospital	2020	Retrospective	23-54 years	5	0	5	Non-severe	5	DM: 1, HTN: 1; High cholesterol: 1; Ulcerative colitis + ileostomy: 1;	• CNIs • Steroids • Antimetabolites	8
									Severe	0			
									Alive	5			
									Dead	0			
2	Lee et al. (9)	USA, Icahn School of Medicine	2020	Retrospective	30-80 years	NA	NA	24	Non-severe	13	HTN: 22; DM: 17; CVD: 10; CKD: 17	• CNIs • Steroids • Antimetabolites	8
									Severe	11			
									Alive	17			
									Dead	7			
3	Loinaz et al. (10)	Spain, Hospital Universitario “12 de Octubre”	2020	Retrospective	46-79 years	12	5	17	Non-severe	16	DM: 6; HTN: 9; Lung disease: 4	• CNIs • Steroids • Antimetabolites mTORi	8
									Severe	1			
									Alive	16			
									Dead	1			
4	Becchetti et al. (6)	Switzerland, Inselspital University Hospital	2020	Prospective	57–70 years	40	17	57	Non-severe	46	CVD: 21; Arterial HTN: 32; DM: 21; Active cancer: 5; COPD: 7; HIV: 1; Kidney insufficiency: 16; Heart failure: 9;	• CNIs • Steroids • Antimetabolites mTORi	8
									Severe	11			
									Alive	50			
									Dead	7			
5	Waisberg et al. (17)	Brazil, Universidade de Sào Paulo	2020	Retrospective	34-69 years	4	0	4	Non-severe	3	HTN 2; DM 1; Obesity 1; Hepatosplenic schistosomiasis 1	• CNIs • Steroids • Antimetabolites	7
									Severe	1			
									Alive	3			
									Dead	1			
6	Webb et al. (12)	UK, University of Oxford	2020	Retrospective	47–66 years	102	49	151	Non-severe	108	Obesity 44; CVD 22; DM 65; Asthma 0 69; COPD 4; CLD 4; HTN 63; Non-liver cancer 8; Stroke 3	• CNIs • Steroids • Antimetabolites mTORi	9
									Severe	43			
									Alive	123			
									Dead	28			
7	Belli et al. (15)	Italy, Niguarda Hospital	2020	Retrospective	55–69 years	171	72	243	Non-severe	206	DM: 94; HTN: 111; CLD: 25; CKD; 49; CAD 17	• CNIs • Steroids • Antimetabolites mTORi	8
									Severe	37			
									Alive	194			
									Dead	49			
8	Felldin et al. (18)	Sweden, Sahlgrenska University Hospital	2020	Retrospective	27–72 years	1	7	8	Non-severe	7	DM: 3; COPD: 1; CKD: 1; Hypothyroid: 1; Sarcoidosis: 1; Polymyalgia rheumatica: 1; CLL: 1	• CNIs • Steroids • Antimetabolites	9
									Severe	1			
									Alive	7			
									Dead	1			

*CAD, coronary artery disease; CKD, chronic kidney disease; CLD, chronic lung disease; CLL, chronic leukocytic leukemia; CVD, cardiovascular disease, CLD, chronic lung disease; CNIs, calcineurin inhibitors; COPD, chronic obstructive pulmonary disease; DM, diabetes mellitus; HTN, hypertension; HIV, human immunodeficiency disease; IS, immunosuppressant; mTORi, mammalian target of rapamycin inhibitor*.

Of the eight included studies, five studies (9, 15–18) compared the mild and severe groups of LT recipients with COVID-19, six studies (6, 10, 12, 16–18) compared the survivor and non-survivor groups of LT recipients with COVID-19. Although we identified eight studies for the inclusion in our analysis, patients from Loinaz et al.'s (10) and Becchetti et al.'s (6) studies seem to be included in Belli et al.'s (15) multicenter study. These studies were only identified to calculate an outcome of interest and were not used collectively in any meta-analysis. In case of the studies from the same institution or authors, we only selected the studies with a greater number of patient samples or those having sufficient data for carrying out a meta-analysis. All the included studies in our meta-analysis were carefully scrutinized for any overlapping authors or institutions. Additionally, the authors were contacted directly through an email in case of any doubt.

### Meta-Analysis

#### Pooled Estimates of the Severity and Mortality in LT Recipients With COVID-19 Under an Immunosuppressive Therapy

Eight studies with a total of 509 LT recipients with COVID-19 were included (6, 9, 10, 12, 15–18). The results showed the prevalence of severity of LT patients with COVID-19 undergoing an immunosuppressive therapy with CNIs (9, 15–18), steroids (9, 15–18), antimetabolites (9, 15–18), mTORi (15), and all the combined immunosuppressive therapies (9, 12, 15–18) were 21.1, 24.7, 26.3, 13.5, and 22.4%, respectively (Supplementary Table 2 and Supplementary Figure 2). Similarly, the incidence of mortality with CNIs (6, 10, 12, 16–18), steroids (6, 10, 12, 16–18), antimetabolites (6, 10, 12, 16–18), mTORi (6, 10, 12), and all the combined immunosuppressive therapies (9, 12, 15–18) were 15, 17.2, 12.1, 8.3, and 19.5%, respectively (Supplementary Table 3 and Supplementary Figure 3).

#### Pooled Estimates of Severity and Mortality in LT Recipients With COVID-19 Based on Comorbidities Under an Immunosuppressive Therapy

Based on comorbidities such as diabetes, hypertension, cardiopulmonary disorders, chronic kidney disease (CKD), age > 60, and obesity, the prevalence of severity of LT patients with COVID-19 undergoing an immunosuppressive therapy were 26.4% (9, 15–18), 37% (9, 15–17), 32.5% (9, 15, 17, 18), 30.2% (9, 15, 18), 24.3% (9, 15, 17, 18), and 31.2% (9, 15–18), respectively. However, the prevalence of severity among LT recipients without comorbidities was only 6% (9, 15–17) (Supplementary Table 4 and Supplementary Figure 4).

Similarly, the incidence of mortality in LT patients with COVID-19 undergoing an immunosuppressive therapy with comorbidities, such as diabetes, hypertension, cardiopulmonary disorders, age > 60, and obesity, were 22.9% (10, 12, 16–18), 21.7% (10, 12, 16, 17), 27.9% (10, 12, 17, 18), 19.4% (6, 10, 17, 18), and 19.5% (10, 12, 16–18), respectively. Nevertheless, the incidence of mortality among LT recipients without comorbidities was only 7.7% (10, 16, 17) (Supplementary Table 5 and Supplementary Figure 5).

#### Comparison Between LT Recipients on an Immunosuppressive Therapy With Non-Severe and Severe COVID-19

**Figure 2:**
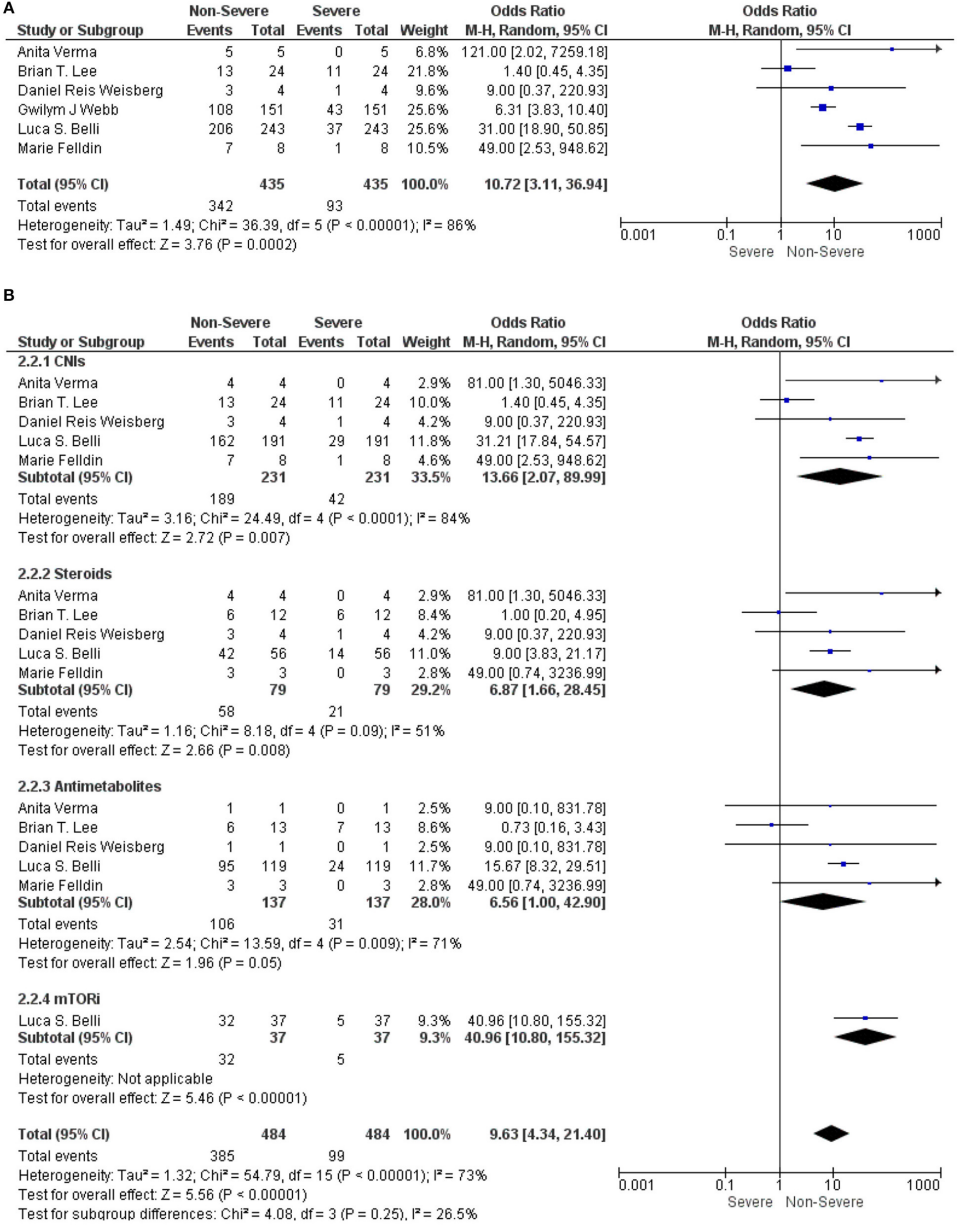
**(A)** Forest plots for an association between the non-severe and severe liver transplantation (LT) recipients with COVID-19 for the overall immunosuppressants (ISs). **(B)** Forest plots for an association between the non-severe and severe LT recipients with COVID-19 with a subgroup analysis for the calcineurin inhibitors (CNIs), steroids, antimetabolites, and mammalian target of rapamycin inhibitors (mTORis) therapy.

We analyzed the immunosuppressive therapies in LT recipients with COVID-19 in between the non-severe and severe groups. A meta-analysis using a random-effect model exhibited that the use of the immunosuppressive therapies in LT recipients with COVID-19 was significantly associated with a non-severe COVID-19 (OR: 10.72, 95% CI: 3.11–36.94; *p* < 0.001) (9, 12, 15–18) for all the immunosuppressive therapies combined (Figure 2A). Similarly, CNIs (OR: 13.66, 95% CI: 2.07–89.99; *p* = 0.007) (9, 15–18), steroids (OR: 6.87, 95% CI: 1.66–28.45; *p* = 0.008) (9, 15–18), and mTORi (OR: 40.96, 95% CI: 10.80–155.32; *p* < 0.001) (15) were also significantly associated with a non-severe COVID-19 in LT recipients. However, antimetabolites were not significantly associated with a non-severe COVID-19 in LT recipients (OR: 6.56, 95% CI: 1.00–42.90; *p* = 0.050) (9, 15–18). Nonetheless, from the trend of a forest plot, antimetabolites seem to be associated with a non-severe COVID-19 in LT recipients. Furthermore, the test for the overall subgroup difference showed no heterogeneity (*I*^2^ = 26.5%; *p* = 0.250) (Figure 2B).

#### Comparison Between the Survivor' and Non-Survivor LT Recipients With COVID-19 Under an Immunosuppressive Therapy

**Figure 3:**
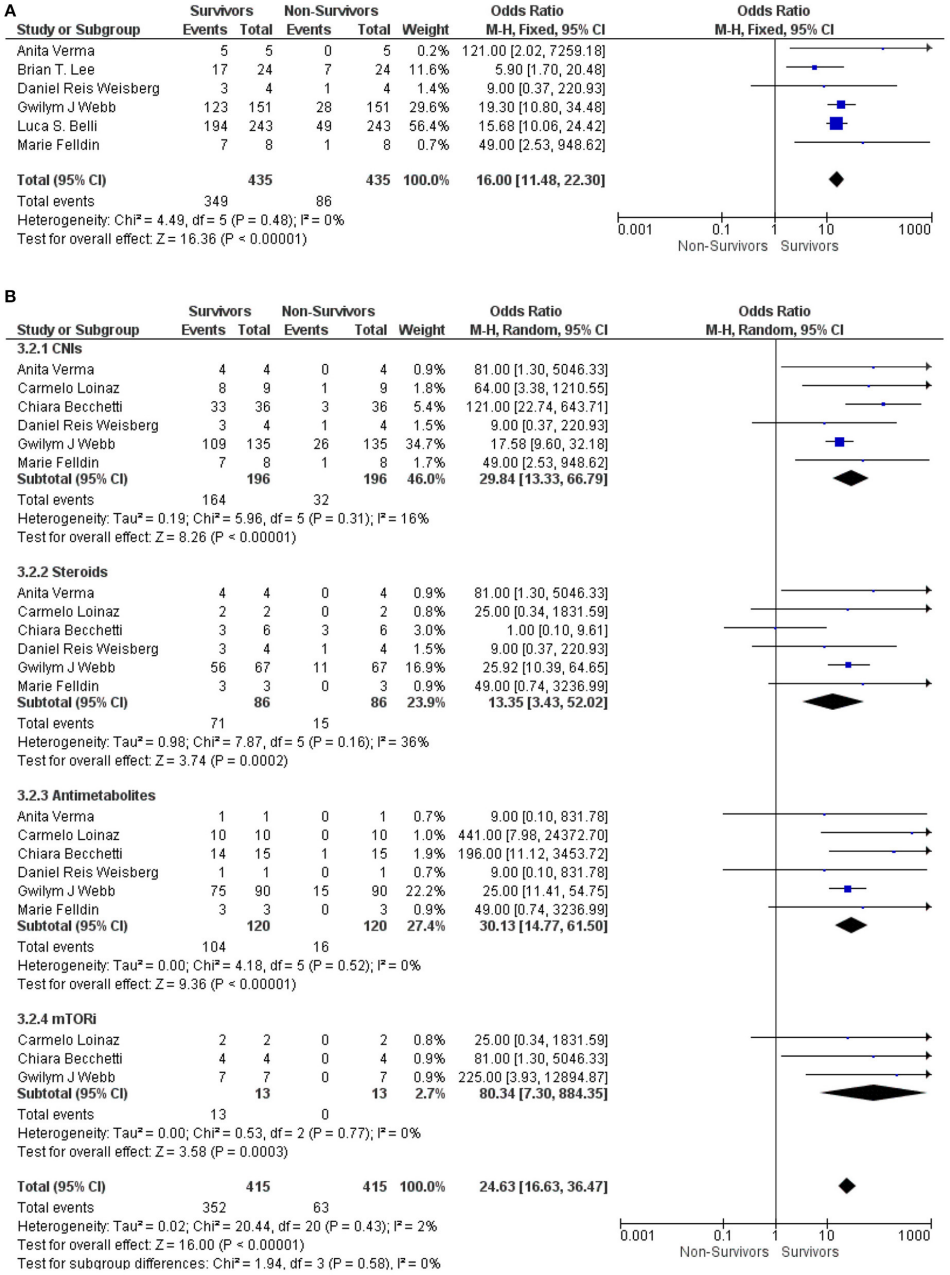
**(A)** Forest plots for an association between the survivors and non-survivors LT recipients with COVID-19 for the overall ISs. **(B)** Forest plots for an association between the survivors and non-survivors LT recipients with COVID-19 with subgroup analysis for the CNIs, steroids, antimetabolites, and mTORis therapy.

The stratified studies according to the immunosuppressive therapies in LT recipients with COVID-19 were further analyzed between the survivor and non-survivor groups. Our meta-analysis found that the use of immunosuppressive therapies in LT recipients with COVID-19 was significantly associated with the survival of the patients (OR: 16.00, 95% CI: 11.48–22.30; *p* < 0.001) (9, 12, 15–18) for all the immunosuppressive therapies combined (Figure 3A). Similarly, CNIs (OR: 29.84, 95% CI: 13.33–66.79; *p* < 0.001) (6, 10, 12, 16–18), steroids (OR: 13.35, 95% CI: 3.43–52.02; *p* < 0.001) (6, 10, 12, 16–18), antimetabolites (OR: 30.13, 95% CI: 14.77–61.50; *p* < 0.001) (6, 10, 12, 16–18), and mTORi (OR: 80.34, 95% CI: 7.30–884.35; *p* < 0.001) (6, 10, 12) were also significantly associated with the survival of LT recipients with COVID-19. Additionally, the test for the overall subgroup difference did not show any heterogeneity (*I*^2^ = 0%; *p* = 0.58) (Figure 3B).

#### Comparison Between LT Recipients on an Immunosuppressive Therapy With Non-Severe and Severe COVID-19 Based on Comorbidities

**Figure 4:**
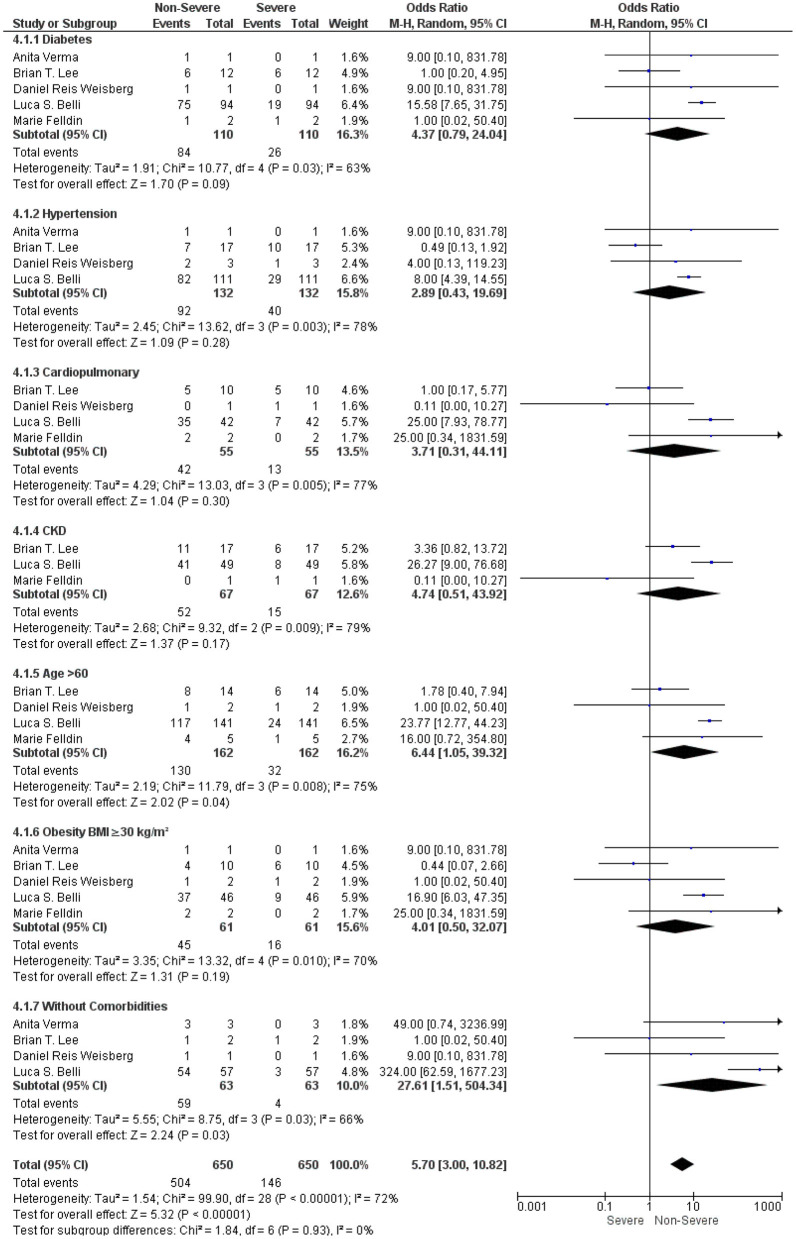
Forest plots for an association between the non-severe and severe LT recipients with COVID-19 based on comorbidities under an immunosuppressive therapy.

We further analyzed the underlying comorbidities in LT recipients with COVID-19 under an immunosuppressive therapy between non-severe and severe groups. We found that comorbidities such as diabetes, hypertension, cardiopulmonary disorders, CKD, and obesity were not significantly associated with a severe COVID-19 in LT recipients with COVID-19 under the immunosuppressive therapies (OR: 4.37, 95% CI: 0.79–24.04; *p* = 0.09) for diabetes (9, 15–18) (OR: 2.89, 95% CI: 0.43–19.69; *p* = 0.28), for hypertension (9, 15–17) (OR: 3.71, 95% CI: 0.31–44.11; *p* = 0.30), for cardiopulmonary disorders (9, 15, 17, 18) (OR: 4.74, 95% CI: 0.51–43.92; *p* = 0.17), for CKD (9, 15, 18), and (OR: 4.01, 95% CI: 0.50–32.07; *p* = 0.19) for obesity (9, 15–18). However, LT recipients with COVID-19 without comorbidities (OR: 27.61, 95% CI: 1.51–504.34; *p* = 0.03) (9, 15–17) and age > 60 (OR: 6.44, 95% CI: 1.05–39.32; *p* = 0.04) (9, 15, 17, 18) were significantly associated with a non-severe COVID-19 under an immunosuppressive therapy. In addition, the test for the overall subgroup difference did not show any heterogeneity (*I*^2^ = 0%; *p* = 0.93) (Figure 4).

#### Comparison Between the Survivor and Non-Survivor LT Recipients With COVID-19 Based on Comorbidities Under an Immunosuppressive Therapy

**Figure 5:**
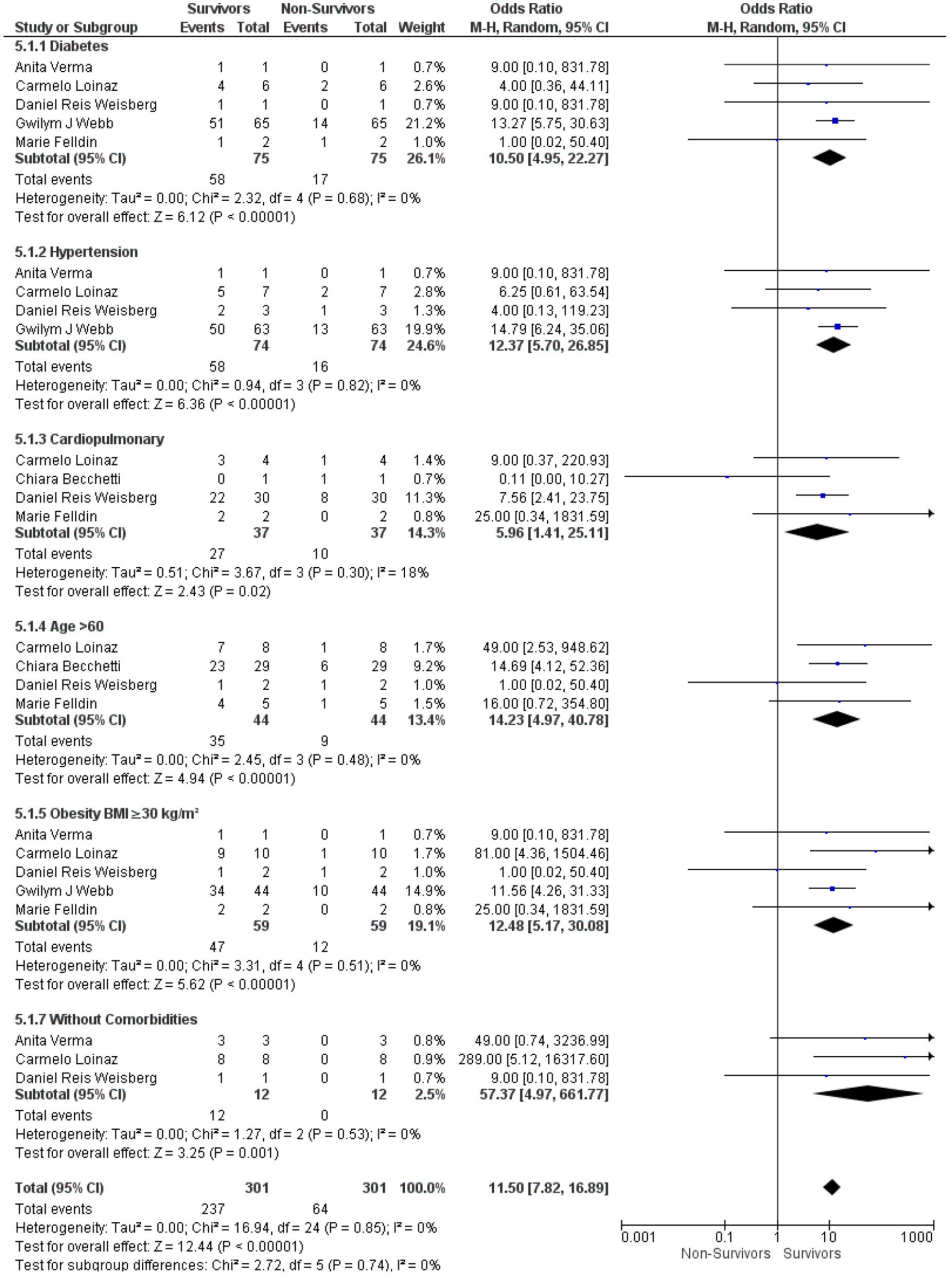
Forest plots for an association between the survivors and non-survivors LT recipients with COVID-19 based on comorbidities under an immunosuppressive therapy.

Then, we analyzed the underlying comorbidities in LT recipients with COVID-19 under an immunosuppressive therapy between the survivor and non-survivor groups. We found that comorbidities, such as diabetes, hypertension, cardiopulmonary disorders, age > 60, and obesity, were significantly associated with the survival outcome in LT recipients with COVID-19 under the immunosuppressive therapies: (OR: 10.50, 95% CI: 4.95–22.27; *p* < 0.001) for diabetes (10, 12, 16–18) (OR: 12.37, 95% CI: 5.70–26.85; *p* < 0.001), for hypertension (10, 12, 16, 17) (OR: 5.96, 95% CI: 1.41–25.11; *p* = 0.02), for cardiopulmonary disorders (6, 10, 17, 18) (OR: 14.23, 95% CI: 4.97–40.78; *p* < 0.001), for age > 60 (6, 10, 17, 18), and (OR: 12.48, 95% CI: 5.17–30.08; *p* < 0.001) for obesity (10, 12, 16–18). Furthermore, LT recipients without comorbidities were also significantly associated with the survival outcome in LT recipients with COVID-19 under the immunosuppressive therapies (OR: 57.37, 95% CI: 4.97–661.77; *p* < 0.001) (10, 16, 17). In addition, the test for the overall subgroup difference did not show any heterogeneity (*I*^2^ = 0%; *p* = 0.74) (Figure 5).

#### Comparison Between LT Recipients on an Immunosuppressive Therapy With Non-Severe and Severe COVID-19 Based on the Time of Transplantation

As LT recipients are on the higher doses of IS in the first year of LT, which is slowly lowered onward as a maintenance dose; thus, as some hypotheses, it may lead to an increase in viral load and a delayed recovery from COVID-19 in these LT recipients during the first year of LT. Secondly, the longer the transplant time, the higher the rates of comorbidity due to chronic immunosuppression, which can also negatively impact the outcomes of LT recipients with COVID-19. Therefore, we further stratified the studies according to the time of transplantation, i.e., >1 and <1 year of LT for LT recipients with COVID-19 and compared between the non-severe and severe groups. Our meta-analysis revealed that the time of transplantation for LT recipients with COVID-19 was not associated with a severe COVID-19. In contrast, it was significantly associated with a non-severe COVID-19 under an immunosuppressive therapy, i.e., (OR: 41.49, 95% CI: 23.97–71.81; *p* < 0.001) (15, 16, 18) for >1 year and (OR: 8.73, 95% CI: 3.06–24.89; *p* < 0.001) (15, 17, 18) for <1 year of LT (Supplementary Figure 6).

#### Comparison Between the Survivor and Non-Survivor LT Recipients With COVID-19 Based on the Time of Transplantation Under an Immunosuppressive Therapy

Similarly, the stratified studies according to the time of transplantation, i.e., >1 year and <1 year of LT for LT recipients with COVID-19 were compared between the survivor and non-survivor groups. Our meta-analysis showed that LT recipients with COVID-19 with the transplantation time >1 year were significantly associated with the survival under an immunosuppressive therapy (OR: 21.68, 95% CI: 12.57–37.39; *p* < 0.001) (10, 12, 16, 18). However, LT recipients with COVID-19 with the transplantation time <1 year were not significantly associated with the survival under an immunosuppressive therapy (OR: 5.69, 95% CI: 0.81–40.16; *p* = 0.08) (10, 17, 18). Yet, from the inclination of a forest plot, <1 year of the transplantation seems to be associated with survival (Supplementary Figure 7).

#### Comparison Between LT Recipients on an Immunosuppressive Therapy With Non-Severe and Severe COVID-19 Based on the Primary Disease for LT

LT recipients with COVID-19 in the included studies were further graded based on the primary diseases for the transplantation, such as liver cancer, decompensated cirrhosis, and others (e.g., acute liver failure, liver metastasis from colorectal cancer, cholestatic liver diseases, etc.) and compared between the non-severe and severe groups. Our meta-analysis demonstrated that primary diseases such as liver cancer and decompensated cirrhosis were significantly associated with a non-severe COVID-19 under an immunosuppressive therapy, i.e., (OR: 16.89, 95% CI: 7.11–40.14; *p* < 0.001) (15, 17) and (OR: 23.34, 95% CI: 12.62–43.19; *p* < 0.001) (15, 17), respectively. However, the other primary diseases apart from the liver cancer and decompensated cirrhosis were not significantly associated with a non-severe COVID-19 under an immunosuppressive therapy (OR: 65.61, 95% CI: 0.80–5,411.52; *p* = 0.06) (15–17). Though, from the course of a forest plot, the other primary diseases seem to be related to a non-severe COVID-19 under an immunosuppressive therapy. In addition, the test for the overall subgroup difference fails to show any heterogeneity (*I*^2^ = 0%; *p* = 0.74) (Supplementary Figure 8).

#### Comparison Between the Survivor and Non-Survivor LT Recipients on an Immunosuppressive Therapy With COVID-19 Based on the Primary Disease for LT

Furthermore, LT recipients with COVID-19 in the included studies were graded on the basis of the primary diseases for the transplantation, i.e., liver cancer, decompensated cirrhosis, and others were compared between the survivor and non-survivor groups. Our meta-analysis revealed that primary diseases such as liver cancer and decompensated cirrhosis were significantly associated with the survival under an immunosuppressive therapy, i.e., (OR: 23.5, 95% CI: 10.04–52.93; *p* < 0.001) (6, 10, 12, 17) and (OR: 29.47, 95% CI: 2.47–351.64; *p* = 0.007) (10, 17), respectively. However, the primary diseases other than the liver cancer and decompensated cirrhosis were insignificantly associated with the survival (OR: 9.16, 95% CI: 0.25–336.67; *p* = 0.23) (6, 10, 12, 16, 17). Yet, from the tendency of a forest plot, other primary diseases seem to be associated with the survival under an immunosuppressive therapy. Notably, the test for the overall subgroup difference did not show any heterogeneity (*I*^2^ = 0%; *p* = 0.87) (Supplementary Figure 9).

## Discussion

The published studies have sufficiently shown that there is a dysregulation in the host immune defense upon SARS-CoV-2 infection, where SARS-CoV-2 infection activates both innate and adaptive immune responses (20, 21). The majority of the COVID-19 patients are invariably observed to have increased pro-inflammatory cytokines, typically leading to hyper-inflammation, i.e., cytokine storm, similar to that in a secondary hemophagocytic lymphohistiocytosis (SHL) (13). The immunosuppression can sometimes be considered as a double-edged sword in the COVID-19 pandemic. Theoretically, the immunosuppression can attenuate the initial inflammatory response in COVID-19, whereas, on the other hand, it may naturally result in an increase in SARS-CoV-2 viral load in LT patients due to the immunosuppressive therapies causing diminished T-cell immunity (8). At present, there are no proper guidelines on the standard doses of the IS in LT recipients with COVID-19 (6, 9, 10). Thus, we aimed to perform a meta-analysis to properly estimate the prognosis of LT recipients on the maintenance IS with COVID-19. Up to the time of writing this meta-analysis, it is the first meta-analysis that compares the severity and mortality of the IS drugs in LT recipients with COVID-19. Our meta-analysis includes eight studies (6, 9, 10, 12, 15–18) with a total of 509 LT recipients with COVID-19.

Apart from being an immunosuppressed group, LT recipients have also a high prevalence of various comorbidities and active malignancy (6); thus a higher rate of severity and mortality can be expected due to COVID-19. As reported in the different recent literature studies, the severity and mortality in LT recipients with COVID-19 are about 19.5–31.5 and 12–18.4%, respectively (6, 7, 9). Consistent with earlier studies, our meta-analysis also found the severity of 22.4% and the mortality of 19.5% in LT recipients with COVID-19. Additionally, our study sufficiently revealed that the IS in LT recipients with COVID-19 was significantly associated with the non-severe disease and survival of the patients. Interestingly, a large cohort affirms a lower rate of mortality (18%) in LT recipients with COVID-19 as compared to that of the matched general population (7). Similarly, an international registry study by Webb et al. also found that LT did not significantly increase the risk of death in patients with COVID-19 [absolute risk difference 1.4% (95% CI: 7.7–10.4)], whereas an increase in age and the presence of comorbidities were associated with death in LT recipients with COVID-19 (12). Besides, it is known that patients with comorbidities pose a substantial risk to develop severe COVID-19 and have a high mortality rate (22). However, our meta-analysis showed that comorbidities such as diabetes, hypertension, cardiopulmonary disorders, CKD, age >60, the duration of LT to the diagnosis of COVID-19, and obesity were not significantly associated with the severity and mortality in LT recipients with COVID-19 under an immunosuppressive therapy. Additionally, the primary diseases for the transplantation were also not significantly associated with the severity and mortality in LT recipients with COVID-19 under an immunosuppressive therapy. For now, we do not know the proper reason behind it; these results might be due the greater numbers of patients with a mild disease in the included studies, or the IS used in LT patients could have protective effects in spite of these patients having comorbidities. Nevertheless, from our pooled analysis, we found that LT recipients with COVID-19 and without comorbidities have a less severe disease and low mortality rate compared to LT recipients with COVID-19 and with comorbidities, i.e., 6 vs. 24.3–37% for severity and 7.7 vs. 19.4–27.9% for mortality.

Colmenero et al. found that CNIs or mTORi was not associated with an unfavorable effect (7). Moreover, tacrolimus (TAC) was found to be an independent protective factor and was associated with a better survival in LT recipients with COVID-19 (7, 15). Furthermore, Cavagna et al. in their 385 consecutive solid organ transplant (SOT) recipients reported that the clinical course of the COVID-19 patients on CNIs was generally mild with no ARDS or infectious complications (23). Interestingly, in the COQUIMA cohort, despite the COVID-19 patients with different comorbidities, cyclosporine was associated with a significant decrease in the mortality in the severe COVID-19 patients (24). With regard to mTORi, some recent studies have advocated mTORi as a therapeutic target for COVID-19 (25, 26). Fascinatingly, metformin also known to inhibit mammalian target of rapamycin (mTOR) have shown a significant mortality benefit in the COVID-19 patients in some recent studies (27, 28). In addition, in an earlier clinical trial, mTORi has also been proven to ameliorate the clinical outcomes of the patients with H1N1 influenza requiring mechanical ventilation (29). This is in line with a few earlier studies; our meta-analysis also showed that CNIs and mTORi were significantly associated with the non-severe disease and survival of LT recipients with COVID-19. From the pooled analysis, we found that CNIs and mTORi had a comparatively lower incidence of the severity compared to steroids and antimetabolites. Moreover, mTORi had the lowest incidence of mortality compared to the other ISs, i.e., 8.3 vs. 15% for CNIs, 17.2% for steroids, and 12.1% for antimetabolites. As it has been seen that COVID-19 has substantially worse clinical outcomes in the older patients. Not surprisingly, in a previous preclinical and clinical study, mTORi has shown to be effective in improving the cardiovascular and renal function (30, 31), reducing the gut dysbiosis (32), reverse immunosenescence (33), and remodeling an immune function in the elderly with a reduction in the infection (33, 34). Therefore, it seems that the results of our meta-analysis have supported the previous studies. However, a larger randomized clinical trial is urgently needed to reach any solid conclusion. At present, there are some ongoing clinical trials (NCT04461340, NCT04341675, NCT04371640, and NCT04409327) to test mTORi as a treatment option in the patients hospitalized with COVID-19.

In a recent comprehensive review, antimetabolites were discontinued or reduced in 84.3% of the cases (35). Other studies on LT recipients with COVID-19 also showed that antimetabolites were held or reduced (6, 15, 17). Strikingly, Colmenero et al. showed that MMF was an independent predictor of a severe COVID-19 (RR = 3.94; *p* = 0.003), particularly at a dose higher than 1,000 mg/day in a large cohort of LT recipients with COVID-19 (7). It is believed that the use of MMF in LT recipients with COVID-19 might exert a synergistic and deleterious effect as most of the COVID-19 patients suffer from severe lymphopenia and thrombocytopenia (36); thus the use of MMF in LT recipients with COVID-19 may further lead to the depletion of T-lymphocytes, with an increase in the CD4^+^/CD8^+^ ratio (37, 38). In addition, MMF has also been associated as a high-risk factor for the development of hypogammaglobulinemia; thus it may pose a higher risk of mortality (5, 39, 40). Therefore, it seems to be a consensus that antimetabolites are suggested to be ceased or at least reduced in a COVID-19 patient. However, our results showed that the incidence of mortality with the antimetabolite drugs was lower compared to the findings of CNIs and steroids. Additionally, our meta-analysis also revealed that MMF was also significantly associated with the survival of LT recipients with COVID-19. Nonetheless, our results revealed that antimetabolites had a higher rate of the severity compared to CNIs, steroids, and mTORi, i.e., 26.3 vs. 21.1, 24.7, and 13.5%, respectively. Yet, the mortality was only 12.1% for antimetabolites, it seems that most of the centers stopped the use of antimetabolites, or changed to the other IS after the worsening condition of the patients. Thus, the use of antimetabolites must be considered carefully in LT recipients with COVID-19.

Because most of the patients with severe COVID-19 have increased in inflammatory mediators; thus corticosteroids have been proposed as an anti-inflammatory drug to prevent or mitigate a systemic inflammatory response. However, there was an additional 4% mortality risk with every 10 mg increase in hydrocortisone-equivalent dosage in COVID-19 patients (41). Meanwhile, studies have also shown the association of steroids with delayed SARS-CoV-2 virus shedding, especially at a higher dosage (42). Besides, COVID-19 is more commonly associated with the coinfections with other viruses or bacteria than it was initially appreciated (43–45). Thus, an intensive steroid therapy may be disadvantageous. A recent phase IIb clinical trial found that a short course of methylprednisolone in the hospitalized COVID-19 patients did not reduce the mortality (44). On the contrary, improved outcomes have been demonstrated by the use of steroids in the RECOVERY trial, suggesting that dexamethasone may reduce the mortality of the severe COVID-19 patients by one-third (46), which indicates the potential benefit of an increase in the corticosteroid in the management of COVID-19. Additionally, a meta-analysis of the clinical trials of critically ill COVID-19 patients found that the administration of systemic corticosteroids was associated with a lower 28-day mortality compared to the usual care or placebo (47). In the context of the steroid use in LT recipients with COVID-19, our study showed that the steroid had a comparatively higher incidence of the severity and mortality compared to CNIs and mTORi. Nevertheless, our meta-analysis also revealed that the steroid was significantly associated with the non-severe disease and survival of LT recipients with COVID-19. Yet, at this point, the potential role of steroids in COVID-19 remains controversial.

**Figure 6:**
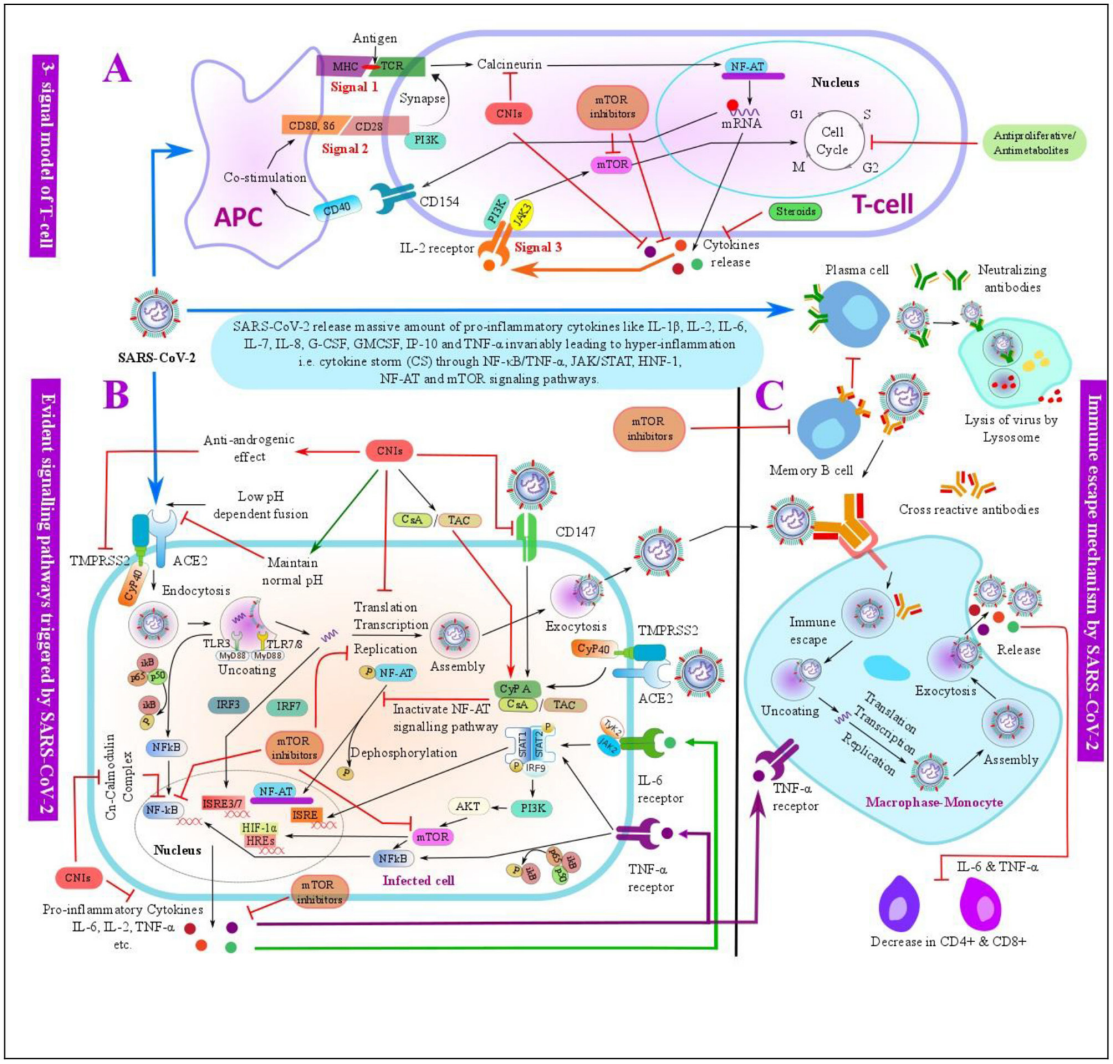
Schematic overview of the evident pathways triggered by severe acute respiratory syndrome coronavirus 2 (SARS-CoV-2) infection and the possible mechanisms of immunosuppressants (ISs) used in the LT with its anti-SARS-CoV-2 effects. **(A)** Three-signal model of the T-cell: SARS-CoV-2 antigen is presented by an antigen-presenting cell (APC) to the T-cell by binding the major histocompatibility complex (MCH) to T-cell receptor (TCR), this further triggers the T-cell signal for an activation and proliferation of the T-cells. Costimulator molecules and its ligand bind at signal 2, which further synapse to TCR at the signal 1. The activation of signals 1 and 2 results in the release of interleukin-2 (IL-2) and other factors. The release of IL-2 further activates an IL-2 receptor on the T-cell surface, which triggers signal 3 for T-cell activation and proliferation. These signals can be inhibited by ISs such as CNIs, antiproliferative/antimetabolites, corticosteroids, and mTORis at various steps. **(B)** Evident pathways triggered by SARS-CoV-2 infection- SARS-CoV-2 may trigger intracellular pathways such as IFN regulatory factor-3 (IRF3), nuclear factor κB/tumor necrosis factor-α (NF-κB/TNF-α), janus kinase/signal transducers and activators of transcription (JAK/STAT), nuclear factor of activated T-cells (NF-AT), hypoxia-inducible factor 1 (HIF-1), and mammalian target of rapamycin (mTOR) signaling pathways within the infected cells, and these pathways can be targeted by the ISs -such as CNIs and mTOR used in LT at various steps. **(C)** Normal immune process of the monocyte-macrophage and possible immune escape mechanism of SARS-CoV-2. Post SARS-CoV-2 infection, SARS-CoV-2 spike protein is bound to the neutralizing antibodies produced by the mature plasma cells, which is then engulfed by the monocyte-macrophage and further decomposed by the lysosome present in the monocyte-macrophage as a normal immune process. SARS-CoV-2 may escape an immune system by the antibody-dependent enhancement (ADE), memory B-cells may secrete a cross-reactive antibody that may bind with SARS-CoV-2 with a weak affinity, which are further engulfed by the monocyte-macrophage. SARS-CoV-2 may get separated from the cross-reactive antibody due to a weak binding consequently leading to an immune escape and further replication and release of the virus along with other cytokines like IL-6 and TNF-α, which may advance to a cytokine storm. In addition, cytokines like IL-6 and TNF-α downregulate CD4+ and CD8+ T-cells. mTOR inhibitors can inhibit the activation of the memory B-cells and therefore downregulate the ADE process. ACE2, angiotensin-converting enzyme 2; AKT, protein kinase B; APC, antigen-presenting cell; CsA, cyclosporine A; CD, cluster of differentiation; CNI, calcineurin inhibitor; CyP, cyclophilin; G-CSF, granulocyte colony-stimulating factor; GM-CSF, granulocyte-macrophage colony-stimulating factor; HIF-1, hypoxia-inducible factor 1; HREs, hormone response elements; IFN, interferon; IgG, immunoglobulin G; IgM, immunoglobulin M; IL, interleukin; IP-10, interferon gamma-induced protein 10; IRF, IFN regulatory factor; ISRE, interferon-stimulated response element; JAK, janus kinase; MHC, major histocompatibility complex; mTOR, mammalian target of rapamycin; NF-AT, nuclear factor of activated T-cells; NF-κB, nuclear factor κB; PI3K, phosphoinositide 3-kinase; SARS-CoV-2, severe acute respiratory syndrome coronavirus 2; STAT, signal transducers and activators of transcription; TAC, tacrolimus; TCR, T-cell receptor; TLRs, toll-like receptors; TMPRSS2, transmembrane protease serine 2; TNF-α, tumor necrosis factor-α.

Our results can also be supported on the background of this basic research. As of now, it is known that SARS-CoV-2 and other coronaviruses use angiotensin-converting enzyme 2 (ACE2) at an acidic cytosolic pH to enter inside the host cells and transmembrane protease serine 2 (TMPRSS2) for the priming and onward transmission (48, 49). It is shown that once the SARS-CoV-2 spike protein binds to the host cells by an ACE2 receptor, it further activates the downstream transduction pathways within the infected cells and may modulate the host immune systems (20, 21, 50). Because the SARS-CoV-2 infection dysregulates and activates immune responses, the maintenance IS used in LT recipients might have a protective role against the SARS-CoV-2 infection (Figure 6).

From *in vitro* studies, CNIs have been found to keep the cytosolic pH at a normal range, thus it may prevent the interaction between SARS-CoV-2 and ACE2 (51). Similarly, CNIs have also shown to display an anti-androgen activity by precisely targeting Cyclophilin (CyP) 40 and thus inhibiting TMPRSS2, which can further inhibit the viral replication (52). Likewise, CNIs may also prevent SARS-CoV-2 entry into the human cells by targeting the CD147 receptors, which facilitate the host cell invasion by SARS-CoV-2 (53). Additionally, CNIs have also been used in the treatment of SHL; hence, it might also mitigate the cytokine storm in COVID-19, and thereby reducing the severity of the disease (13).

Likewise, SARS-CoV-2 infection-related cytokine storm and disease progression are also believed to be associated with an antibody-dependent enhancement (ADE). ADE is a phenomenon of the virus infection in which preexisting cross-reactive antibody enhances virus entry and replication (54). This phenomenon has been observed in various viruses, including the SARS-CoV, MERS, ebola, and dengue viruses (54, 55). Nonetheless, mTOR inhibitors are found to mitigate ADE by selectively inhibiting the memory B-cells, and thereby reducing the production of cross-reactive antibodies (55, 56). Thus, it can be speculated that mTOR inhibitors can also prevent ADE-related disease severity in COVID-19. Additionally, mTOR inhibitors may constrain the secretion of pro-inflammatory cytokines, such as IL-17 and IFN-γ, inhibit the hyperactivation of the CD8^+^ T-cells, and may maintain Treg functions to reduce the cytokine storm in COVID-19 (57). Likewise, mTOR inhibitors increase the performance of the memory T-cells and limit the replication of various viruses, such as cytomegalovirus, Epstein–Barr, and HIV (58).

Kato et al. demonstrated that MPA targeted the coronaviral papain-like protease and sharply reduced the replication of SARS-CoV-2 at EC_50_ of 0.87 μM (59). In addition, MMF was found to inhibit the replication of the human parainfluenza virus type 2 at 2 μg/ml through inhibiting the viral genome RNA, messenger RNA (mRNA), and protein syntheses (60). Therefore, a very low concentration of MMF seems to be sufficient against SARS-CoV-2.

Taking all these into consideration, a randomized controlled trial mainly with CNIs and mTORi alone or in a combination in LT recipients with COVID-19 could be of a great interest.

## Limitations

In spite of relatively high-quality studies included in our meta-analysis, this meta-analysis has various shortcomings. Firstly, there is a potential publication bias, only English language studies were included in this meta-analysis, so the quality of outcomes might have been compromised to a certain extent. Secondly, the management of IS in LT recipients with COVID-19 has been heterogeneous among different studies. Though we analyzed and calculated the effect of a single IS agent by stratifying the patients, according to the single molecule; however, most of the patients were more than one IS agent. To minimize this bias; therefore, we also calculated the effect of an overall IS in LT recipients with COVID-19. Thirdly, substantial data on the incidence of rejection and doses of the IS were lacking. So, we were unable to conduct a meta-analysis of graft rejection and IS dose effects, in the case of a withdrawal or reduction in the doses of IS. Finally, the criteria for the classification of COVID-19 based on the severity were not the same among the studies; this might have contributed to some heterogeneity in our meta-analysis. Nonetheless, this meta-analysis is still of a great significance for assessing the effect of the overall IS and also comparing the outcomes of different ISs in LT recipients with COVID-19 based on the severity and mortality. Thus, it may prove beneficial for the clinicians to choose an appropriate immunosuppressive regimen for LT recipients with COVID-19, so that the management of LT recipients with COVID-19 can be done effectively, hence reducing morbidity and mortality.

## Conclusion

In conclusion, LT recipients with COVID-19 undergoing immunosuppressive therapies are not significantly associated with the severity and mortality and might have a protective role. Thus, taking the risk of organ rejection into key consideration, a complete withdrawal of the IS may not be wise. However, MMF might be discontinued or replaced from an immunosuppressive regimen with the CNIs or mTORis in some selected LT recipients with COVID-19, depending upon the severity of the disease.

## Data Availability Statement

The original contributions presented in the study are included in the article/Supplementary Materials, further inquiries can be directed to the corresponding author/s.

## Author Contributions

DY, QL, and TL involved in the concept and design of this study and critically revised the manuscript. DY, VA, and QL contributed to the acquisition and interpretation of the data. DY, VA, and QL involved in drafting of the manuscript. DY contributed to the figure concept and design. All authors approved the final manuscript.

## Funding

This study was supported by National Natural Science Foundation of China (81830089, U20A20378 to TL, and 81771713 to QL), National Key Research and Development Program (2019YFC1316000 to TL), Zhejiang Provincial Traditional Chinese Medicine Key Discipline Project (2017-XK-A38 to TL), and Zhejiang Provincial Natural Science Foundation of China (2019C03019 to TL and LR18H030001 to QL).

## Conflict of Interest

The authors declare that the research was conducted in the absence of any commercial or financial relationships that could be construed as a potential conflict of interest.

## Publisher's Note

All claims expressed in this article are solely those of the authors and do not necessarily represent those of their affiliated organizations, or those of the publisher, the editors and the reviewers. Any product that may be evaluated in this article, or claim that may be made by its manufacturer, is not guaranteed or endorsed by the publisher.

## Abbreviations

ACE2: angiotensin-converting enzyme 2
ADE: antibody-dependent enhancement
AKT: protein kinase B
APC: antigen-presenting cell
ARDS: acute respiratory distress syndrome
AZA: azathioprine
CD: cluster of differentiation
CKD: chronic kidney disease
CNI: calcineurin inhibitor
COVID-19: coronavirus disease 2019
CsA: cyclosporine A
CyP: cyclophilin
G-CSF: granulocyte colony-stimulating factor
GM-CSF: granulocyte-macrophage colony-stimulating factor
HIF-1
hypoxia-inducible factor 1
HREs: hormone response elements
ICU: intensive care unit
IFN: interferon
IgG: immunoglobulin G
IgM: immunoglobulin M
IL: interleukin
IP-10: interferon gamma-induced protein 10
IRF: IFN regulatory factor
IRS: immune reconstitution syndrome
IS: immunosuppressant
ISRE: interferon-stimulated response element
JAK: janus kinase
LT: liver transplant
MERS: middle east respiratory syndrome
MeSH: medical subject headings
MHC: major histocompatibility complex
MMF: mycophenolate mofetil
MPA: mycophenolic acid
mTORi: mammalian target of rapamycin inhibitor
NF-AT: nuclear factor of activated T-cells
NF-κB: nuclear factor κB
NOS: Newcastle–Ottawa scale
OR: odds ratio
PI3K: phosphoinositide 3-kinase
PRISMA: preferred reporting items for systematic reviews and meta-analyses
SARS: severe acute respiratory syndrome
SARS-CoV-2: severe acute respiratory syndrome coronavirus 2
SHL: secondary hemophagocytic lymphohistiocytosis
SOT: solid organ transplant
SpO2: pulse oxygen saturation
STAT: signal transducers and activators of transcription
TAC: tacrolimus
TCR: T-cell receptor
TLRs: toll-like receptors
TMPRSS2: transmembrane protease serine 2
TNF-α: tumor necrosis factor-α.
