# Supplementary material for: Immunosuppressants in Liver Transplant Recipients With Coronavirus Disease 2019: Capability or Catastrophe?—A Systematic Review and Meta-Analysis
Source: Front Med (Lausanne). 2021 Nov 11;8:756922. doi: 10.3389/fmed.2021.756922 (PMC8632009; doi:10.3389/fmed.2021.756922)
Supplement: Supplementary file 1 [file Data_Sheet_1.PDF]

Supplementary figure 1: Funnel plots for ruling out the publication bias.

Supplementary figure 1.1 Funnel plot for an association of the severity of the LT recipients with COVID-19 undergoing overall IS therapy.

Supplementary figure 1.2 Funnel plot for an association of the mortality of the LT recipients with COVID-19 undergoing overall IS therapy.

Supplementary figure 1.3 Funnel plot for an association of the severity of the LT recipients with COVID-19 undergoing IS therapy with CNIs, steroids, antimetabolites and mTORi.

Supplementary figure 1.4 Funnel plot for an association of the mortality of the LT recipients with COVID-19 undergoing IS therapy with CNIs, steroids, antimetabolites and mTORi.

Supplementary figure 1.5 Funnel plot for an association of the severity of the LT recipients with COVID-19 with diabetes, hypertension, cardiopulmonary disorders, chronic kidney disease, obesity and without comorbidities undergoing IS therapy.

Supplementary figure 1.6 Funnel plot for an association of the mortality of the LT recipients with COVID-19 with diabetes, hypertension, cardiopulmonary disorders, chronic kidney disease, obesity and without comorbidities undergoing IS therapy.

Supplementary figure 1.7 Funnel plots for an association between the non-severe and severe LT recipients with COVID-19 on the basis of the time of transplant.

Supplementary figure 1.8 Funnel plots for an association between the survivors and non-survivors LT recipients with COVID-19 on the basis of the time of transplant.

Supplementary figure 2: Pooled estimates of the severity in the LT recipients with COVID-19 on the basis of IS therapy.

Supplementary figure 2.1 Pooled estimates of the severity for the overall IS therapy in the LT recipients with COVID-19.

Supplementary figure 2.2 Pooled estimates of the severity in the LT recipients with COVID-19 undergoing IS therapy with the CNIs.

Supplementary figure 2.3 Pooled estimates of the severity in the LT recipients with COVID-19 undergoing IS therapy with the steroid.

Supplementary figure 2.4 Pooled estimates of the severity in the LT recipients with COVID-19 undergoing IS therapy with the antimetabolites.

Supplementary figure 3: Pooled estimates of the mortality in the LT recipients with COVID-19 on the basis of IS therapy.

Supplementary figure 3.1 Pooled estimates of the mortality for the overall IS therapy in the LT recipients with COVID-19.

Supplementary figure 3.2 Pooled estimates of the mortality in the LT recipients with COVID-19 undergoing IS therapy with the CNIs.

Supplementary figure 3.3 Pooled estimates of the mortality in the LT recipients with COVID-19 undergoing IS therapy with the steroid.

Supplementary figure 3.4 Pooled estimates of the mortality in the LT recipients with COVID-19 undergoing IS therapy with the antimetabolites.

Supplementary figure 3.5 Pooled estimates of the mortality in the LT recipients with COVID-19 undergoing IS therapy with the mTORi.

Supplementary figure 4: Pooled estimates of the severity in the LT recipients with COVID-19 on the basis of comorbidities.

Supplementary figure 4.1 Pooled estimates of the severity in the LT recipients with COVID-19 with diabetes.

Supplementary figure 4.2 Pooled estimates of the severity in the LT recipients with COVID-19 with hypertension.

Supplementary figure 4.3 Pooled estimates of the severity in the LT recipients with COVID-19 with cardiopulmonary disorders.

Supplemental figure 4.4 Pooled estimates of the severity in the LT recipients with COVID-19 with CKD.

Supplemental figure 4.5 Pooled estimates of the severity in the LT recipients with COVID-19 with age> 60.

Supplementary figure 4.6 Pooled estimates of the severity in the LT recipients with COVID-19 with obesity.

Supplementary figure 4.7 Pooled estimates of the severity in the LT recipients with COVID-19 without comorbidities.

Supplementary figure 5 Pooled estimates of the mortality in the LT recipients with COVID-19 on the basis of comorbidities.

Supplementary figure 5.1 Pooled estimates of the mortality in the LT recipients with COVID-19 with diabetes.

Supplementary figure 5.2 Pooled estimates of the mortality in the LT recipients with COVID-19 with hypertension.

Supplementary figure 5.3 Pooled estimates of the mortality in the LT recipients with COVID-19 with cardiopulmonary disorders.

Supplementary figure 5.4 Pooled estimates of the mortality in the LT recipients with COVID-19 with age> 60.

Supplementary figure 5.5 Pooled estimates of the mortality in the LT recipients with COVID-19 with obesity.

Supplementary figure 5.6 Pooled estimates of the mortality in the LT recipients with COVID-19 without comorbidities.

Supplementary figure 6 Forest plots for an association between the non-severe and severe LT recipients with COVID-19 on the basis of the time of transplant.

Supplementary figure 7 Forest plots for an association between the survivors and non-survivors LT recipients with COVID-19 on the basis of the time of transplant.

Supplementary table 1: Newcastle-Ottawa scale (NOS) of an included studies.

Supplementary table 2: Pooled estimates of the severity in the LT recipients with COVID-19 undergoing IS therapy.

Supplementary table 3: Pooled estimates of the mortality in the LT recipients with COVID-19 undergoing IS therapy.

Supplementary table 4: Pooled estimates of the severity in the LT recipients with COVID-19 on the basis of comorbidities.

Supplementary table 5: Pooled estimates of the mortality in the LT recipients with COVID-19 on the basis of comorbidities.

Supplementary figure 1: Funnel plots for ruling out the publication bias.

Supplementary figure 1.1 Funnel plot for an association of the severity of the LT recipients with COVID-19 undergoing overall IS therapy.

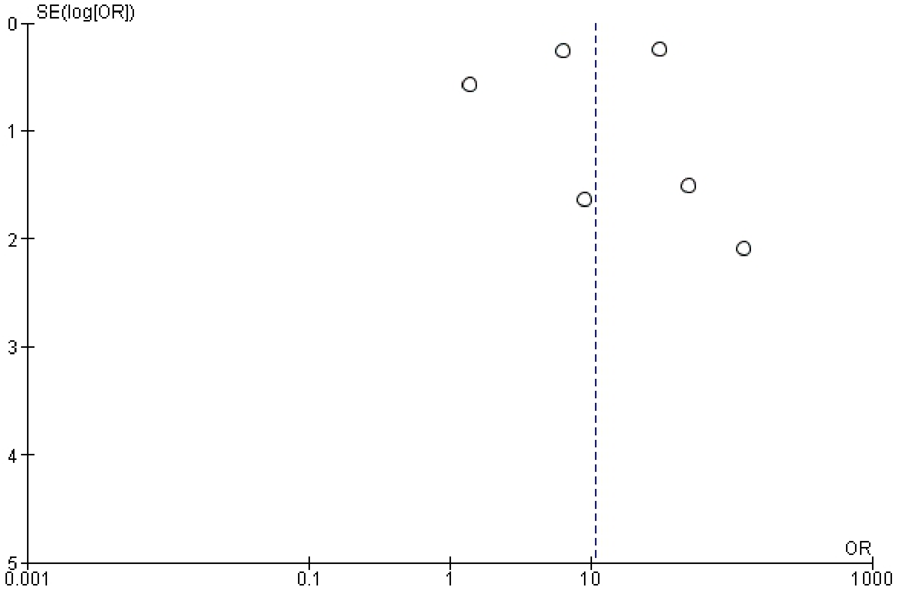

Supplementary figure 1.2 Funnel plot for an association of the mortality of the LT recipients with COVID-19 undergoing overall IS therapy.

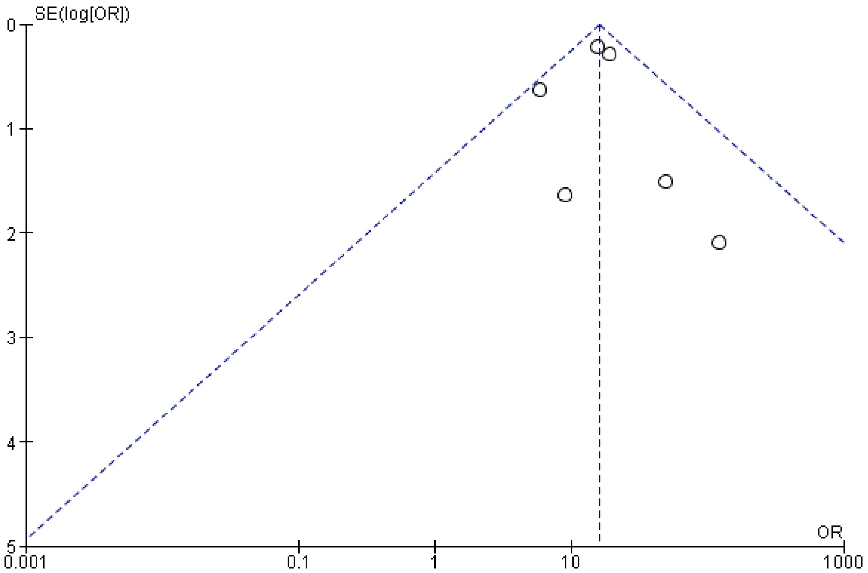

Supplementary figure 1.3 Funnel plot for an association of the severity of the LT recipients with COVID-19 undergoing IS therapy with CNIs, steroids, antimetabolites and mTORi.

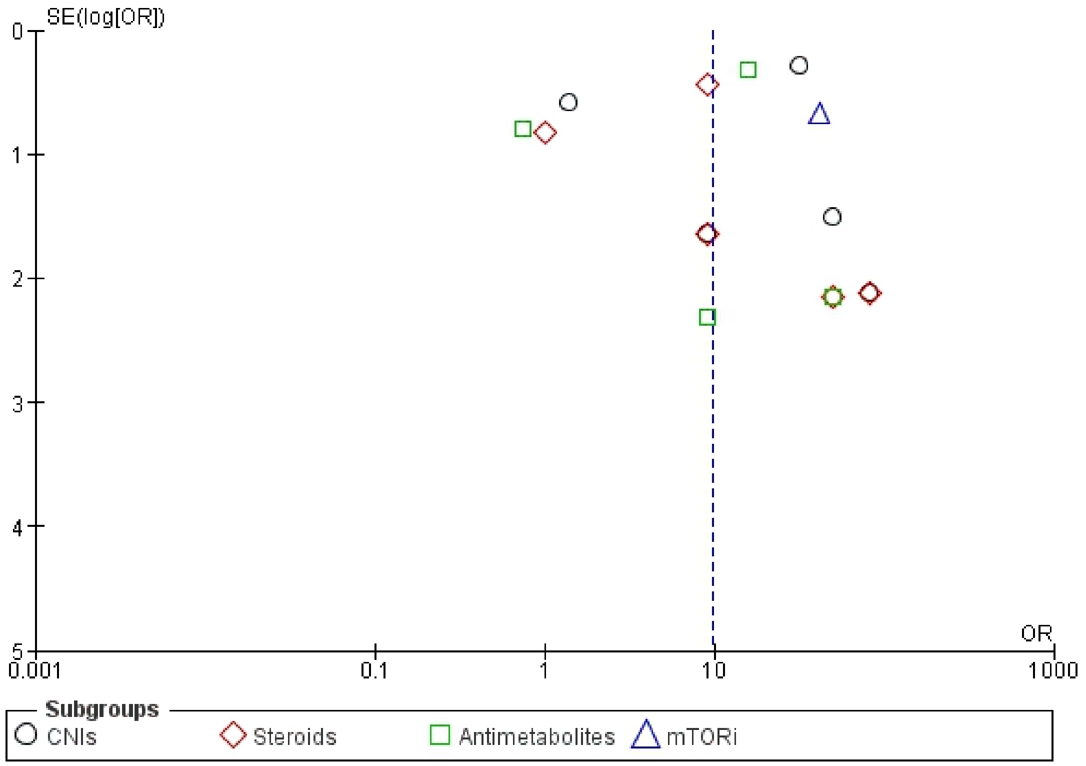

Supplementary figure 1.4 Funnel plot for an association of the mortality of the LT recipients with COVID-19 undergoing IS therapy with CNIs, steroids, antimetabolites and mTORi.

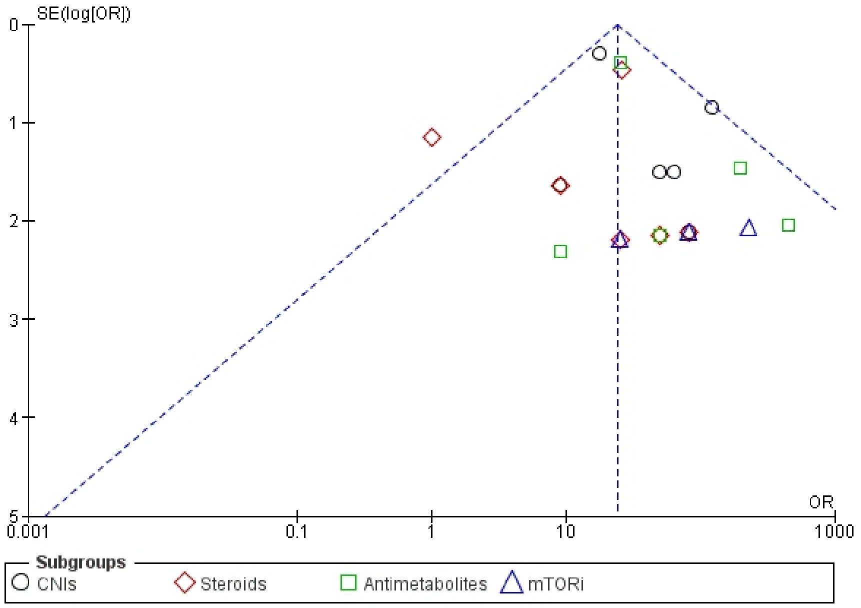

Supplementary figure 1.5 Funnel plot for an association of the severity of the LT recipients with COVID-19 with diabetes, hypertension, cardiopulmonary disorders, chronic kidney disease, obesity and without comorbidities undergoing IS therapy.

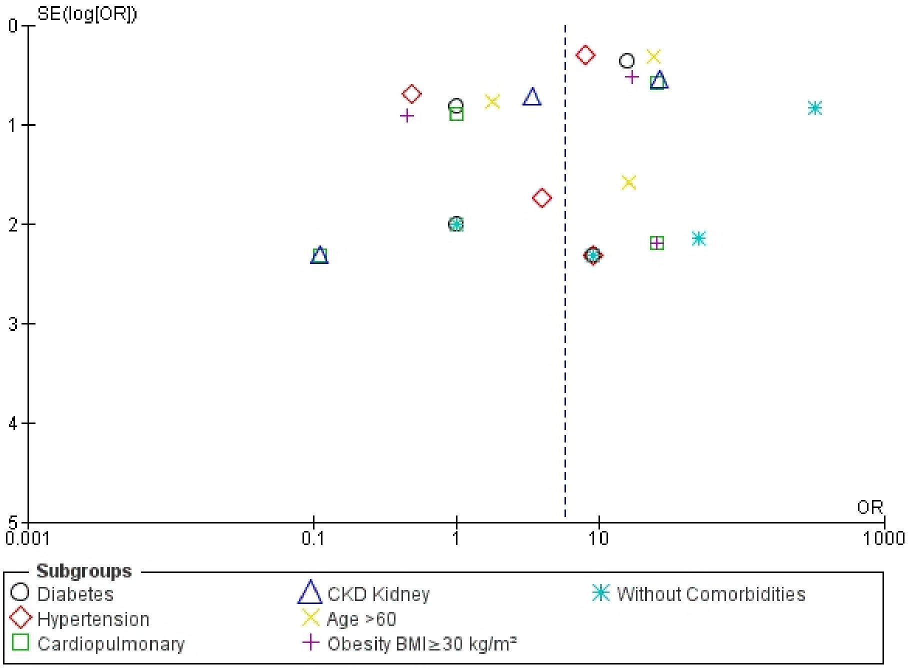

Supplementary figure 1.6 Funnel plot for an association of the mortality of the LT recipients with COVID-19 with diabetes, hypertension, cardiopulmonary disorders, chronic kidney disease, obesity and without comorbidities undergoing IS therapy.

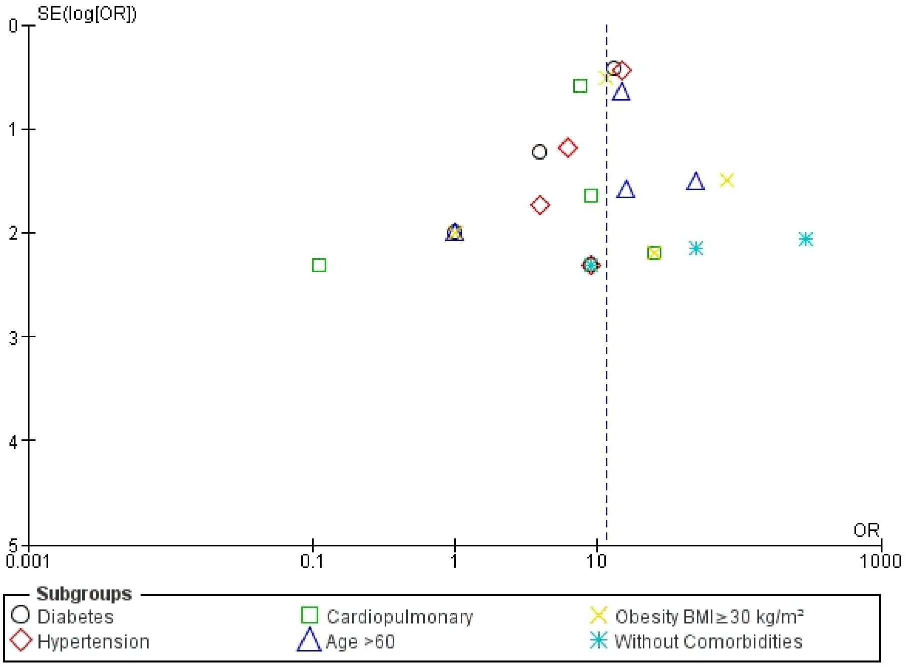

Supplementary figure 1.7 Funnel plots for an association between the non-severe and severe LT recipients with COVID-19 on the basis of the time of transplant.

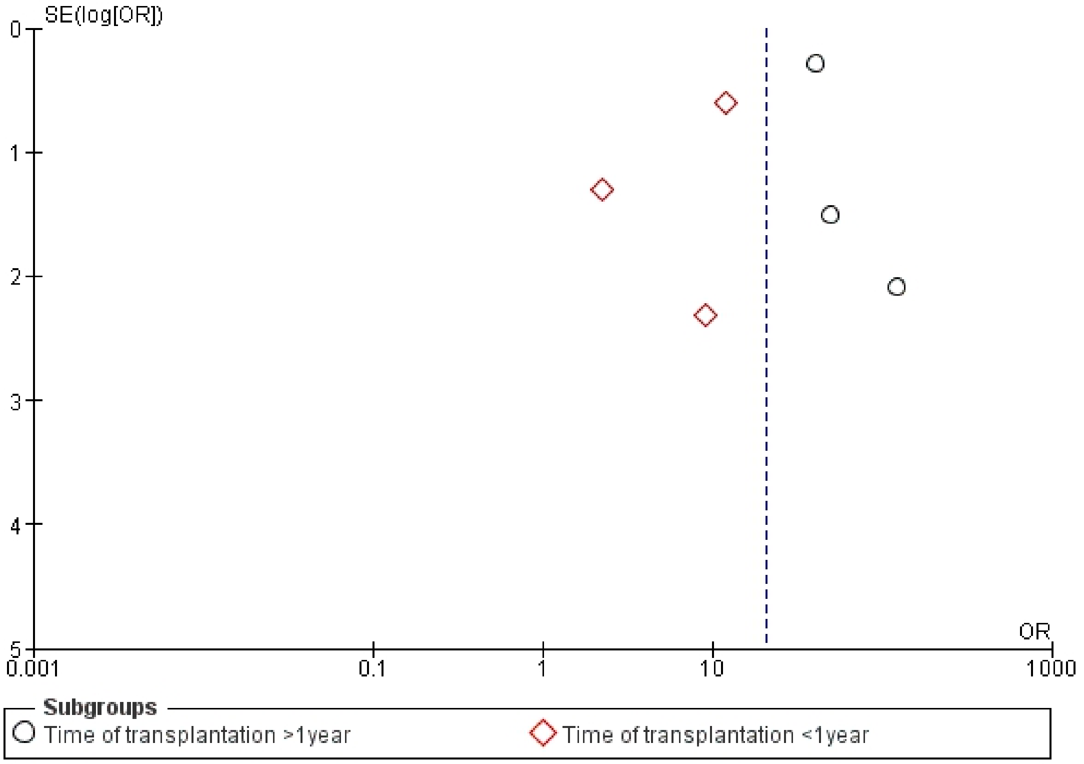

Supplementary figure 1.8 Funnel plots for an association between the survivors and non-survivors LT recipients with COVID-19 on the basis of the time of transplant.

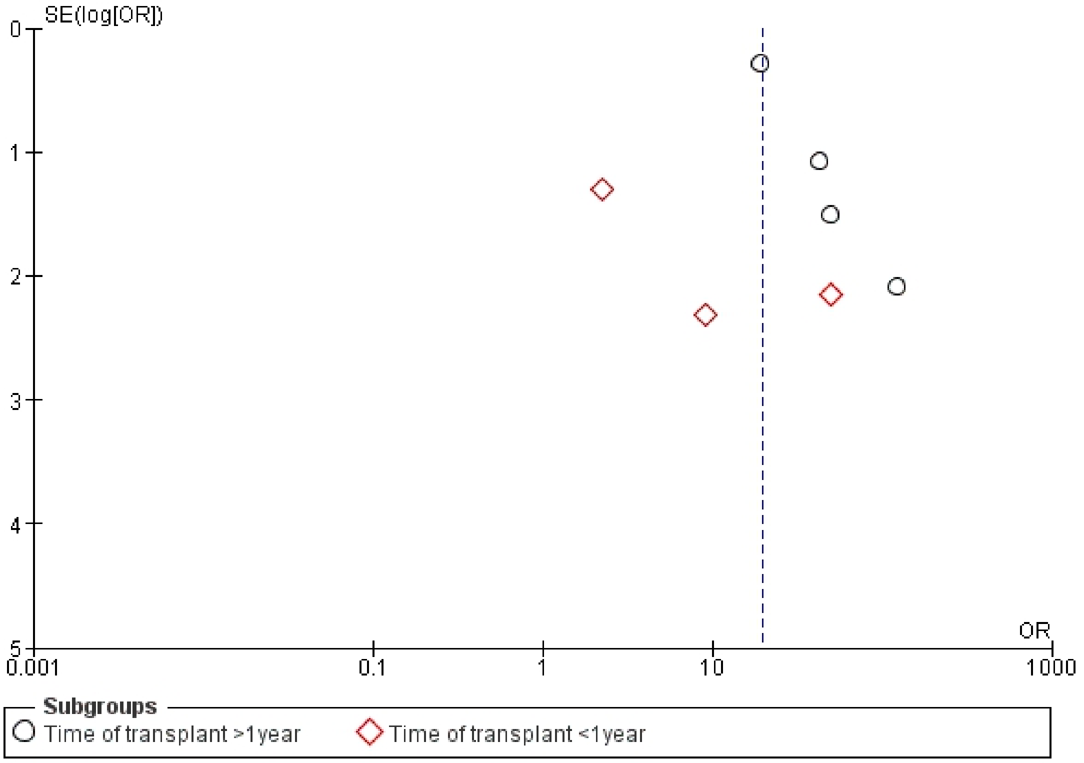

Supplementary figure 1.9 Funnel plots for an association between the non-severe and severe LT recipients with COVID-19 on the basis of the primary disease for the LT.

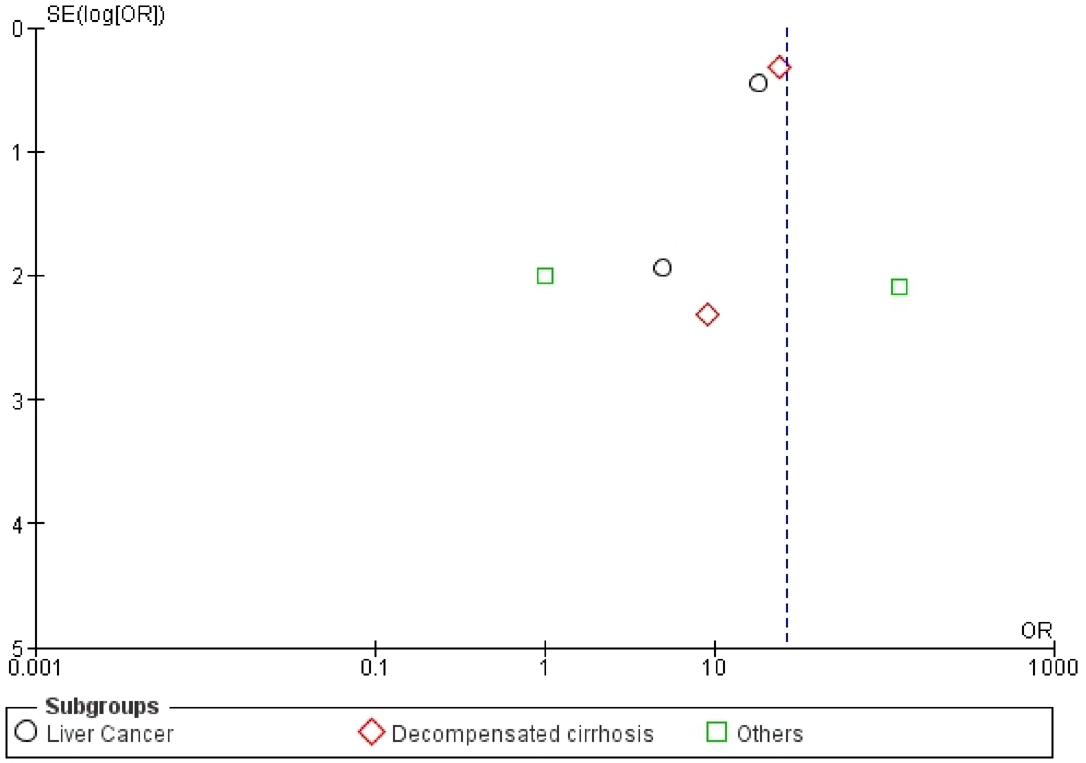

Supplementary figure 1.10 Funnel plots for an association between the survivors and non-survivors LT recipients with COVID-19 on the basis of the primary disease for the LT.

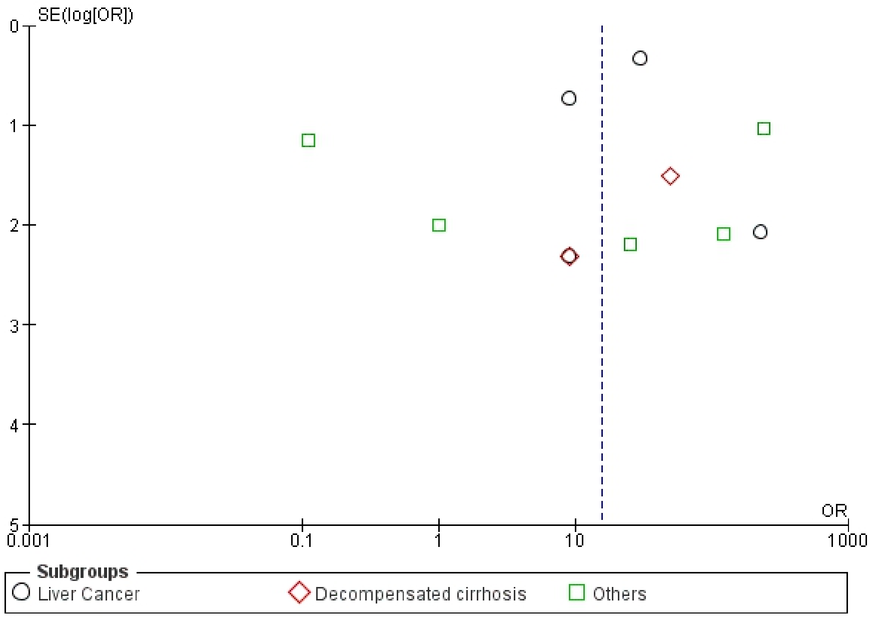

Supplementary figure 2: Pooled estimates of the severity in the LT recipients with COVID-19 on the basis of IS therapy.

Supplementary figure 2.1 Pooled estimates of the severity for the overall IS therapy in the LT recipients with COVID-19.

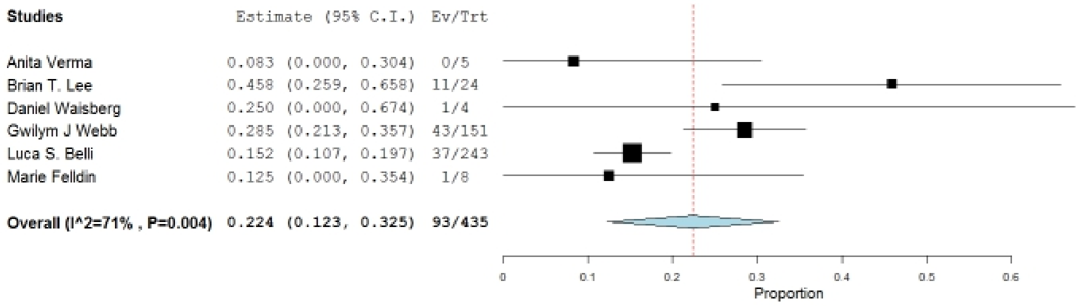

Supplementary figure 2.2 Pooled estimates of the severity in the LT recipients with COVID-19 undergoing IS therapy with the CNIs.

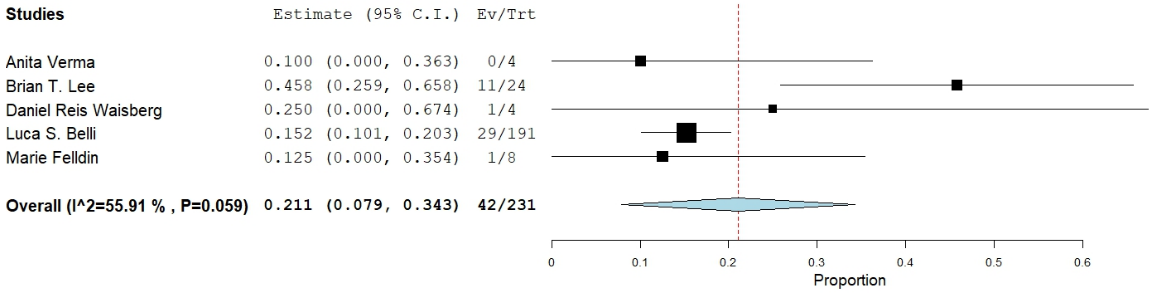

Supplementary figure 2.3 Pooled estimates of the severity in the LT recipients with COVID-19 undergoing IS therapy with the steroid.

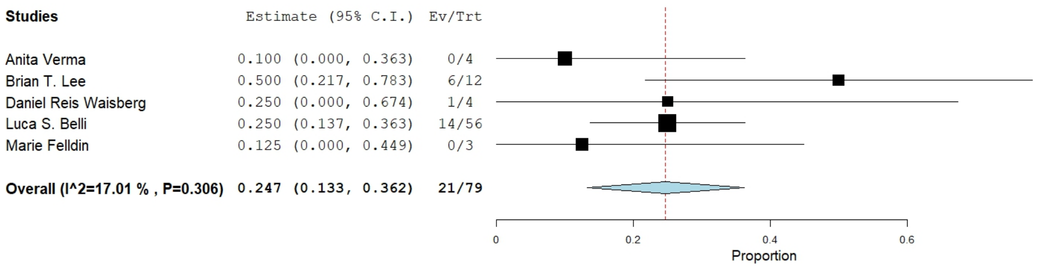

Supplementary figure 2.4 Pooled estimates of the severity in the LT recipients with COVID-19 undergoing IS therapy with the antimetabolites.

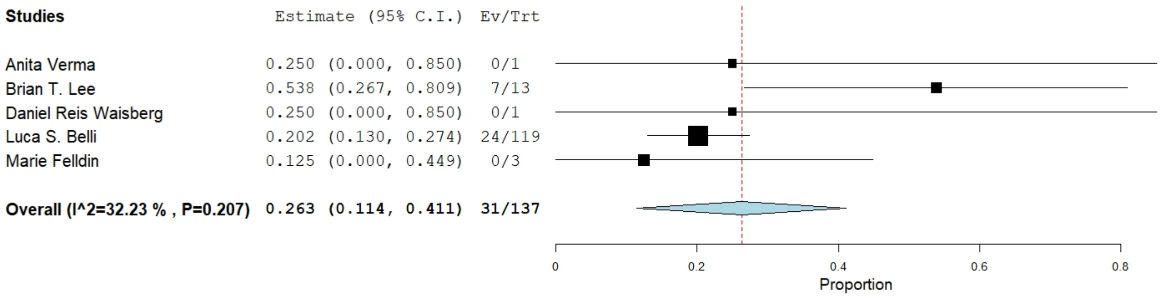

Supplementary figure 3: Pooled estimates of the mortality in the LT recipients with COVID-19 on the basis of IS therapy.

Supplementary figure 3.1 Pooled estimates of the mortality for the overall IS therapy in the LT recipients with COVID-19.

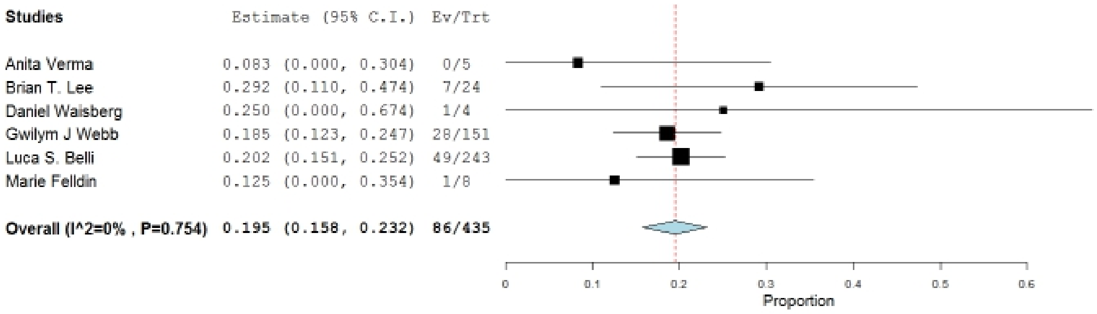

Supplementary figure 3.2 Pooled estimates of the mortality in the LT recipients with COVID-19 undergoing IS therapy with the CNIs

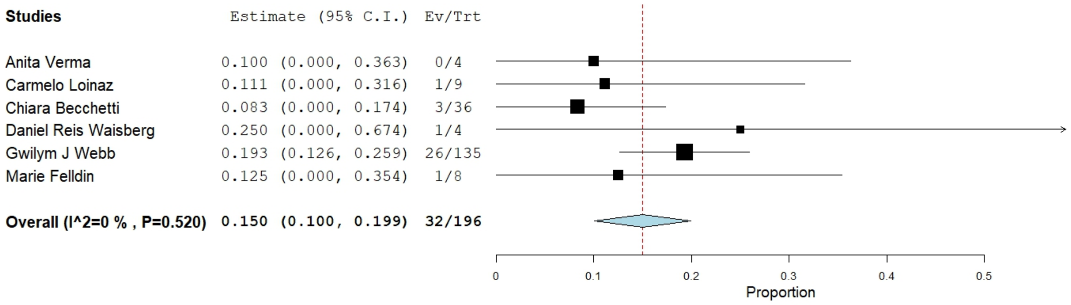

Supplementary figure 3.3 Pooled estimates of the mortality in the LT recipients with COVID-19 undergoing IS therapy with the steroid.

.

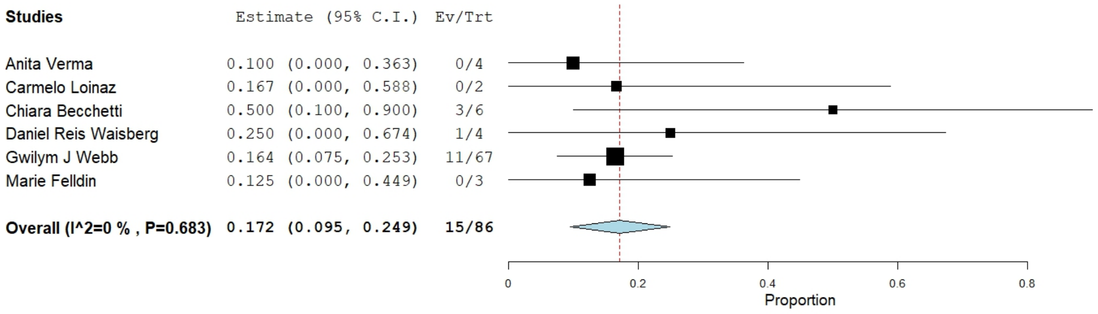

Supplementary figure 3.4 Pooled estimates of the mortality in the LT recipients with COVID-19 undergoing IS therapy with the antimetabolites.

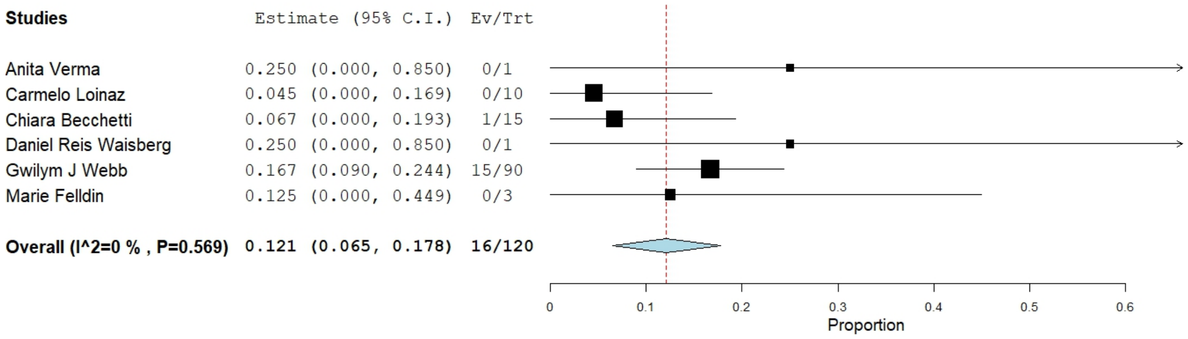

Supplementary figure 3.5 Pooled estimates of the mortality in the LT recipients with COVID-19 undergoing IS therapy with the mTORi.

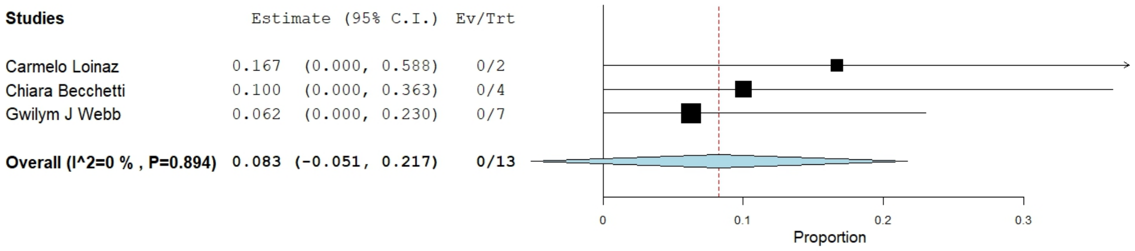

Supplementary figure 4: Pooled estimates of the severity in the LT recipients with COVID-19 on the basis of comorbidities.

Supplementary figure 4.1 Pooled estimates of the severity in the LT recipients with COVID-19 with diabetes.

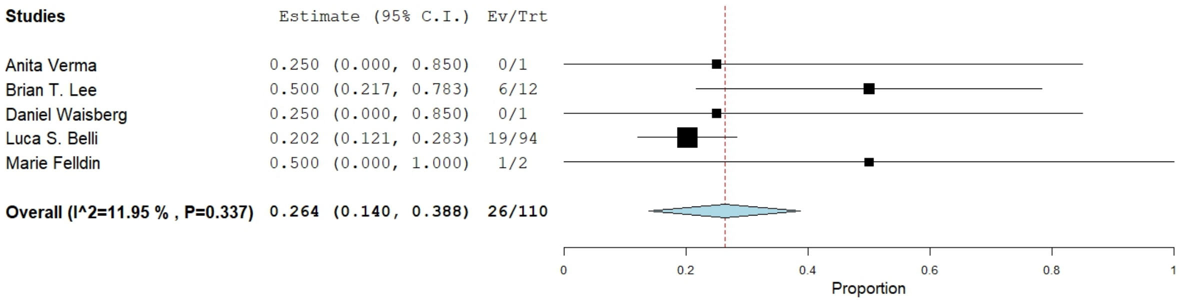

Supplementary figure 4.2 Pooled estimates of the severity in the LT recipients with COVID-19 with hypertension.

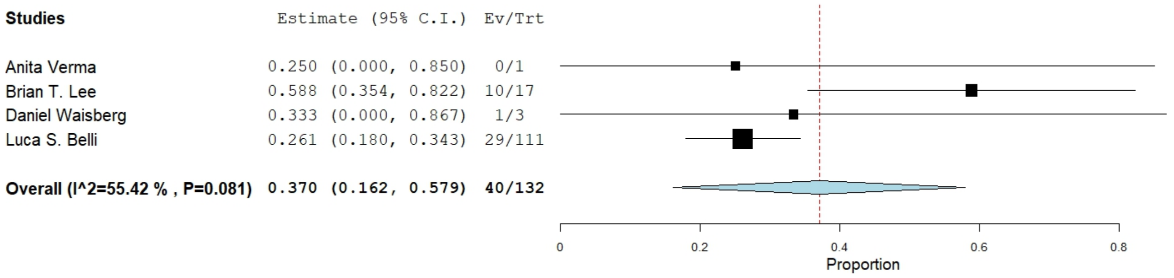

Supplementary figure 4.3 Pooled estimates of the severity in the LT recipients with COVID-19 with cardiopulmonary disorders.

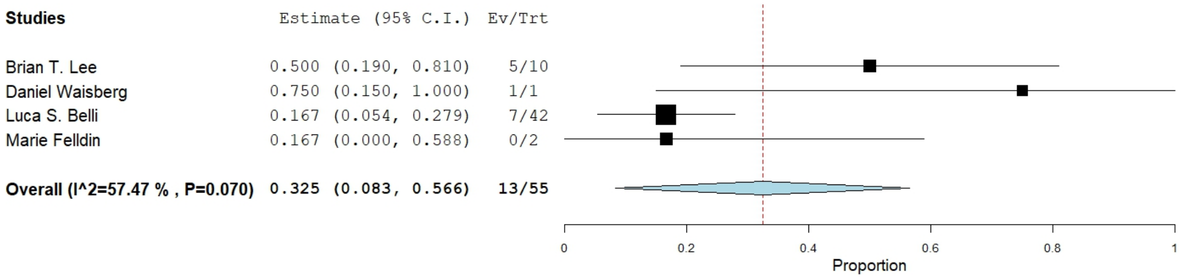

Supplemental figure 4.4 Pooled estimates of the severity in the LT recipients with COVID-19 with CKD.

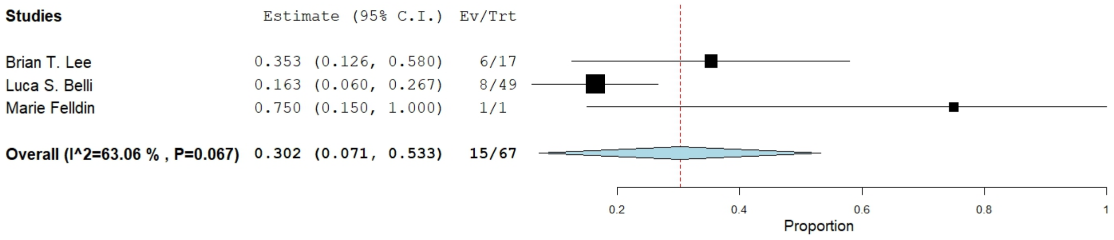

Supplemental figure 4.5 Pooled estimates of the severity in the LT recipients with COVID-19 with age> 60.

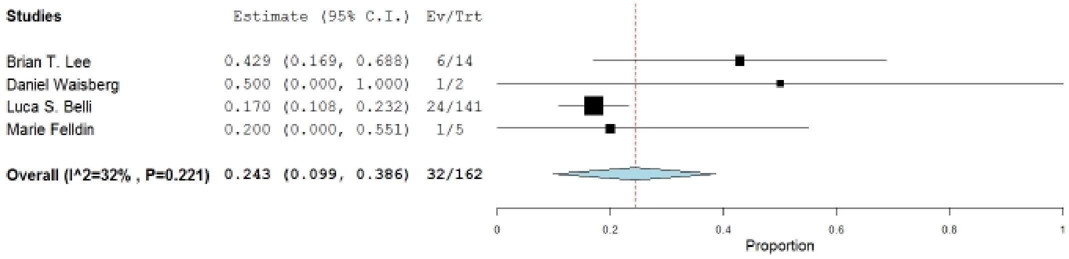

Supplementary figure 4.6 Pooled estimates of the severity in the LT recipients with COVID-19 with obesity.

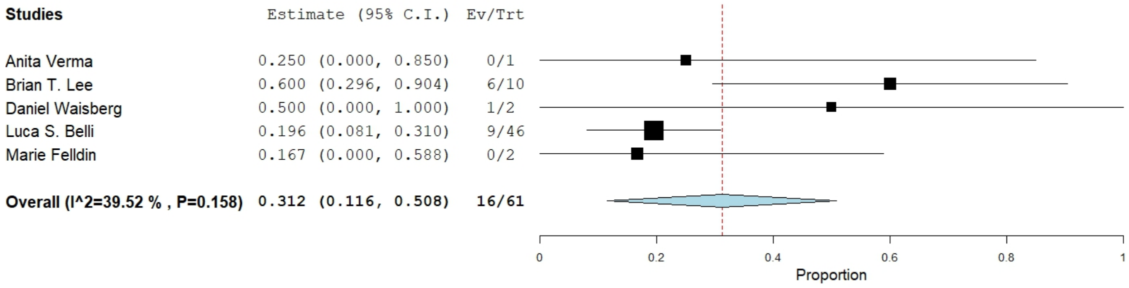

Supplementary figure 4.7 Pooled estimates of the severity in the LT recipients with COVID-19 without comorbidities.

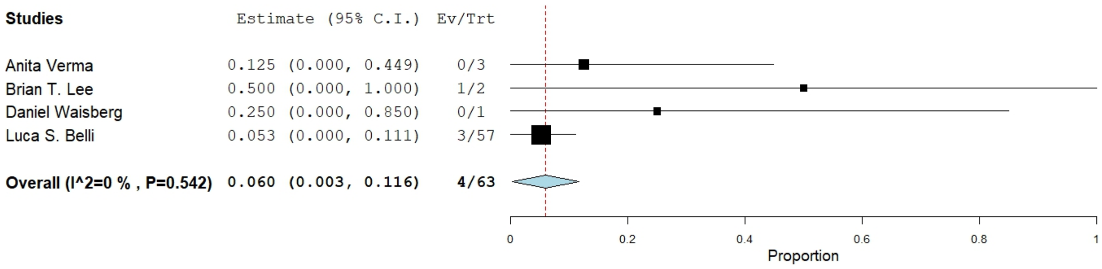

Supplementary figure 5 Pooled estimates of the mortality in the LT recipients with COVID-19 on the basis of comorbidities.

Supplementary figure 5.1 Pooled estimates of the mortality in the LT recipients with COVID-19 with diabetes.

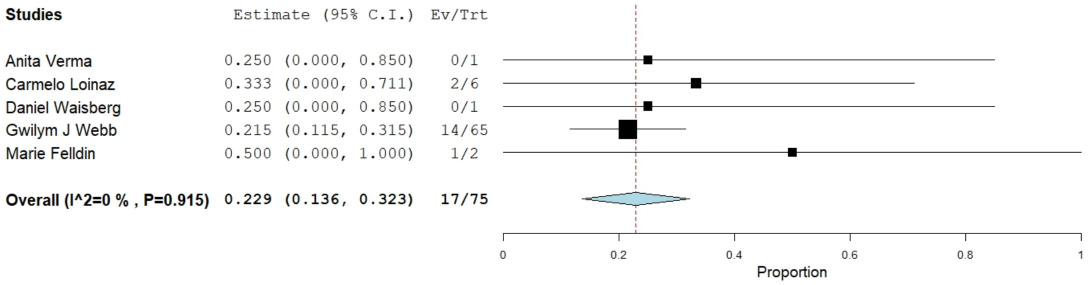

Supplementary figure 5.2 Pooled estimates of the mortality in the LT recipients with COVID-19 with hypertension.

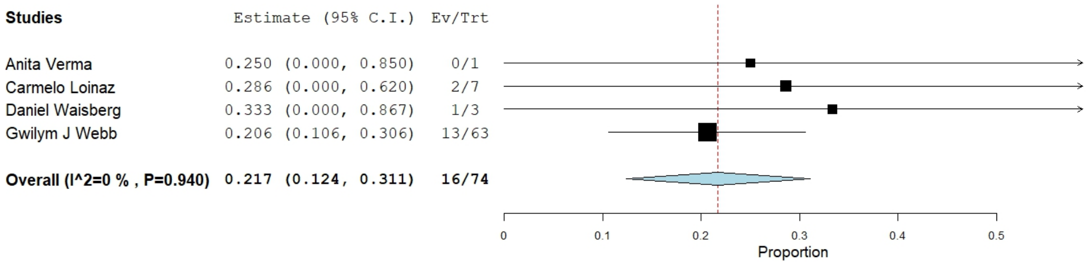

Supplementary figure 5.3 Pooled estimates of the mortality in the LT recipients with COVID-19 with cardiopulmonary disorders.

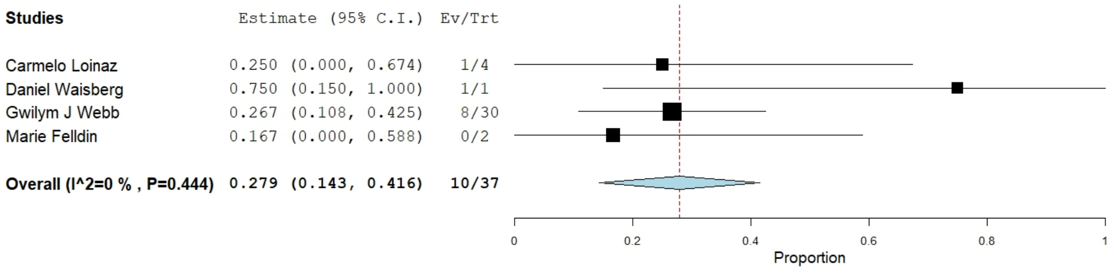

Supplementary figure 5.4 Pooled estimates of the mortality in the LT recipients with COVID-19 with age> 60.

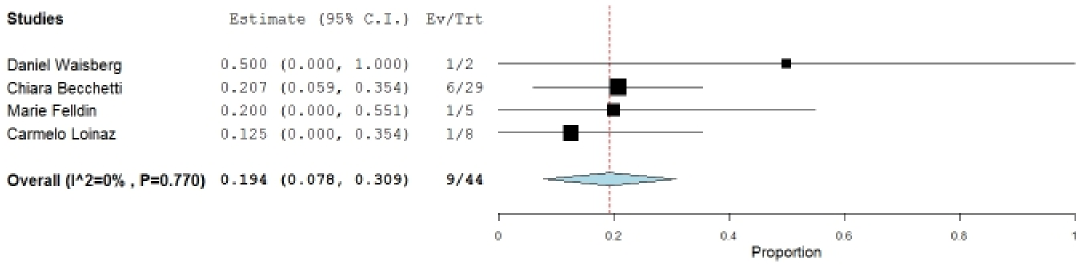

Supplementary figure 5.5 Pooled estimates of the mortality in the LT recipients with COVID-19 with obesity.

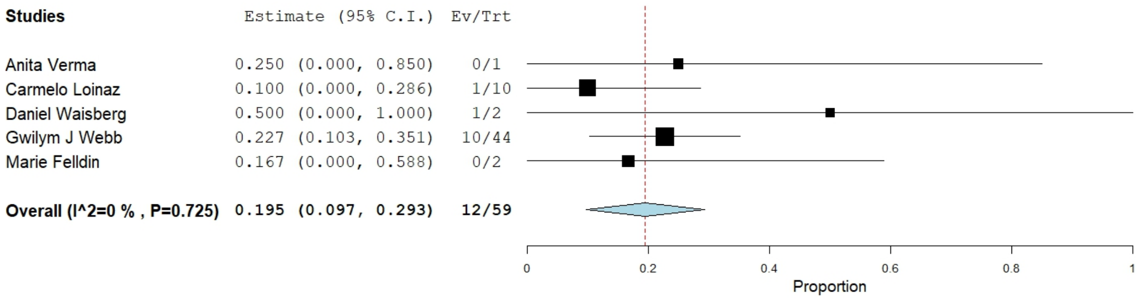

Supplementary figure 5.6 Pooled estimates of the mortality in the LT recipients with COVID-19 without comorbidities.

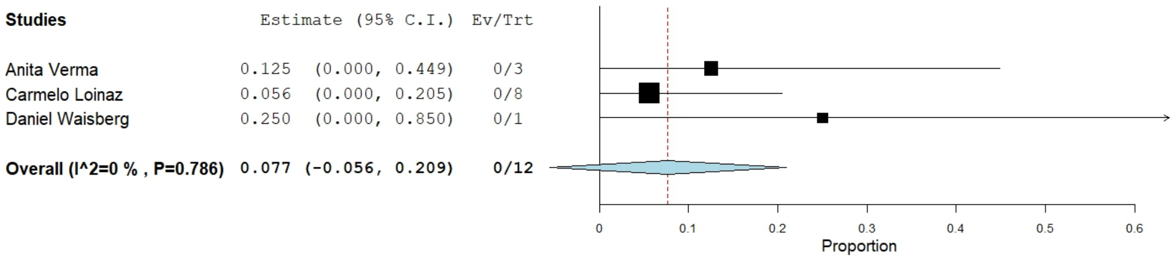

Supplementary figure 6 Forest plots for an association between the non-severe and severe LT recipients with COVID-19 on the basis of the time of transplant.

.

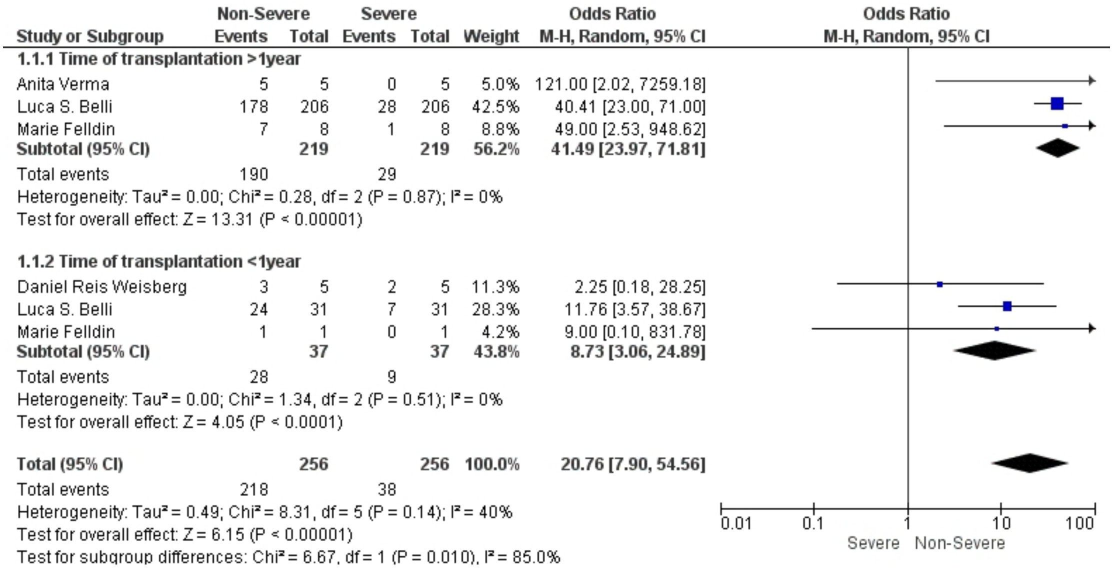

Supplementary figure 7 Forest plots for an association between the survivors and non-survivors LT recipients with COVID-19 on the basis of the time of transplant.

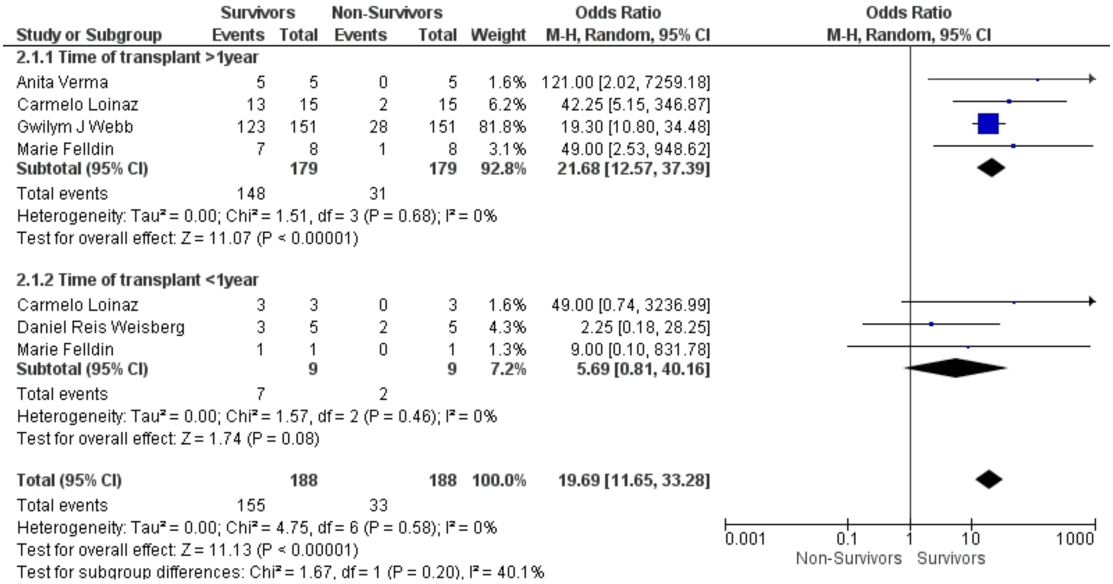

Supplementary figure 8 Forest plots for an association between the non-severe and severe LT recipients with COVID-19 on the basis of the primary disease for the LT.

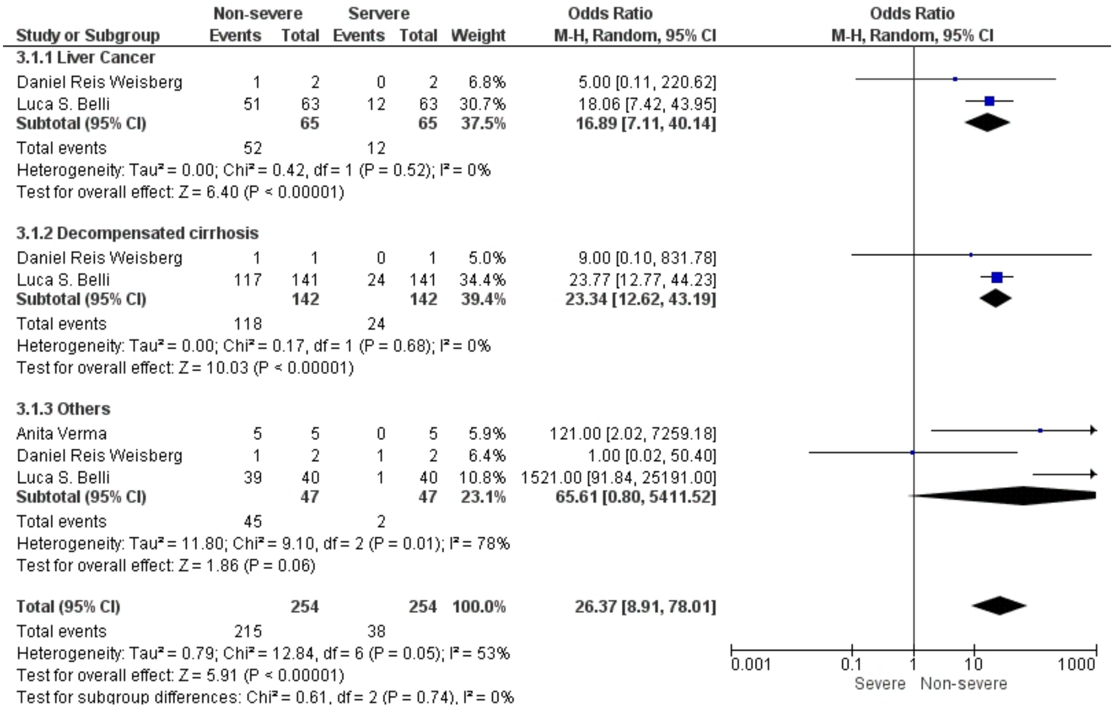

Supplementary figure 9 Forest plots for an association between the survivors and non-survivors LT recipients with COVID-19 on the basis of the primary disease for the LT.

	Survivors		Non-survivors			Odds Ratio
Study or Subgroup	Events	Total	Events	Total	Weight	M-H, Random, 95% CI
**4.1.1 Liver Cancer**						
Carmelo Loinaz	7	7	0	7	6.7%	225.00 [3.93, 12894.87]
Chiara Becchetti	15	20	5	20	13.8%	9.00 [2.15, 37.66]
Daniel Reis Weisberg	1	1	0	1	5.8%	9.00 [0.10, 831.78]
Gwilym J Webb	121	143	22	143	15.7%	30.25 [15.91, 57.51]
**Subtotal (95% CI)**		**171**		**171**	**42.0%**	**23.05 [10.04, 52.93]**
Total events	144		27			
Heterogeneity: Tau² = 0.16; Chi² = 3.62, df = 3 (P = 0.31); I² = 17%						
Test for overall effect: Z = 7.40 (P < 0.00001)						
**4.1.2 Decompensated cirrhosis**						
Carmelo Loinaz	7	8	1	8	9.2%	49.00 [2.53, 948.62]
Daniel Reis Weisberg	1	1	0	1	5.8%	9.00 [0.10, 831.78]
**Subtotal (95% CI)**		**9**		**9**	**15.0%**	**29.47 [2.47, 351.64]**
Total events	8		1			
Heterogeneity: Tau² = 0.00; Chi² = 0.38, df = 1 (P = 0.54); I² = 0%						
Test for overall effect: Z = 2.67 (P = 0.007)						
**4.1.3 Others**						
Anita Verma	5	5	0	5	6.6%	121.00 [2.02, 7259.18]
Carmelo Loinaz	2	2	0	2	6.2%	25.00 [0.34, 1831.59]
Chiara Becchetti	31	33	2	33	12.0%	240.25 [31.80, 1815.07]
Daniel Reis Weisberg	1	2	1	2	7.0%	1.00 [0.02, 50.40]
Gwilym J Webb	2	8	6	8	11.2%	0.11 [0.01, 1.07]
**Subtotal (95% CI)**		**50**		**50**	**43.0%**	**9.16 [0.25, 336.67]**
Total events	41		9			
Heterogeneity: Tau² = 13.95; Chi² = 27.75, df = 4 (P < 0.0001); I² = 86%						
Test for overall effect: Z = 1.20 (P = 0.23)						
**Total (95% CI)**		**230**		**230**	**100.0%**	**15.50 [3.95, 60.86]**
Total events	193		37			
Heterogeneity: Tau² = 3.00; Chi² = 34.94, df = 10 (P = 0.0001); I² = 71%						
Test for overall effect: Z = 3.93 (P < 0.0001)						
Test for subgroup differences: Chi² = 0.29, df = 2 (P = 0.87), I² = 0%						

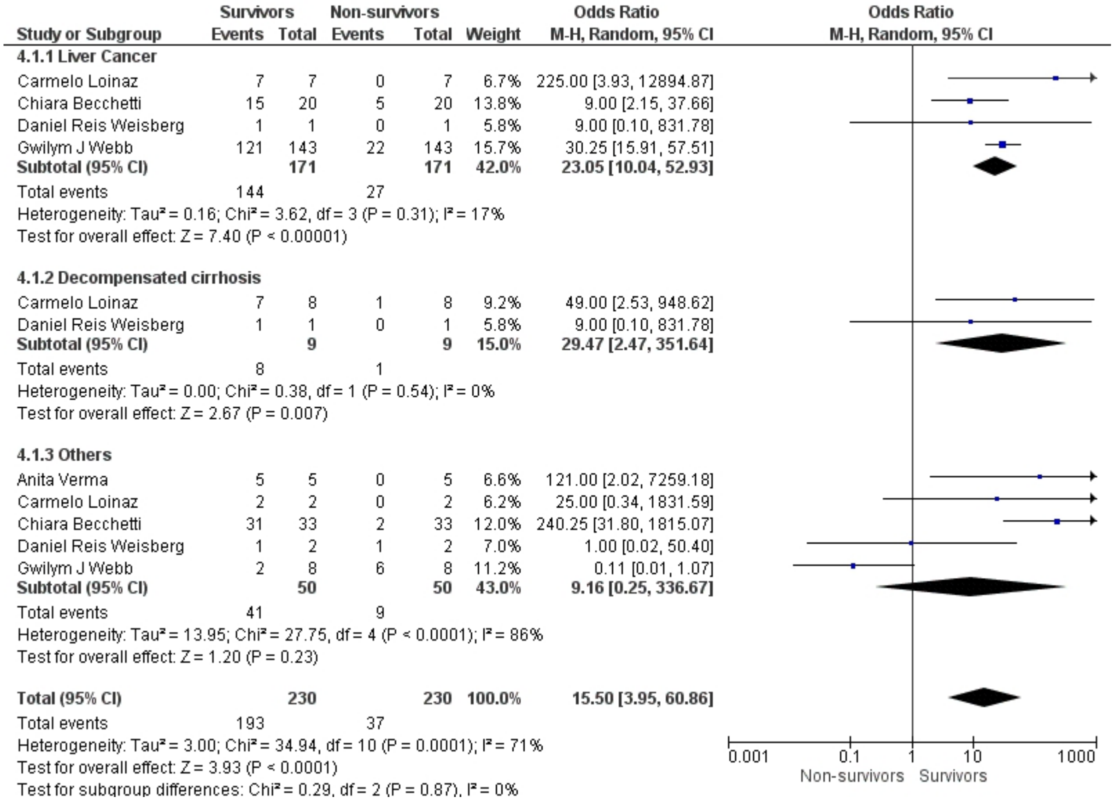

Supplementary table 1: Newcastle-Ottawa scale (NOS) of an included studies.

Study	Selection				Comparison		Outcome			Total quality score
	Representativeness of exposed cohort	Selection of nonexposed cohort	Ascertainment of exposure	Demonstration that outcome of interest not present at start of study	Adjust for the most important risk factors	Adjust for other risk factors	Assessment of outcome	Follow-up length	Loss to follow-up rate	
Anita Verma(16)	1	0	1	1	1	1	1	1	1	8
Brian T. Lee(9)	1	0	1	1	1	1	1	1	1	8
Carmelo Loinaz(10)	1	0	1	1	1	1	1	1	1	8
Chiara Becchetti(6)	1	0	1	1	1	1	1	1	1	8
Daniel Reis Waisberg(17)	1	0	1	1	1	0	1	1	1	7
Gwilym J Webb(12)	1	1	1	1	1	1	1	1	1	9
Luca S. Belli(15)	1	0	1	1	1	1	1	1	1	8
Marie Felldin(18)	1	1	1	1	1	1	1	1	1	9

High quality studies: > 7 points
Moderate quality studies: 4-6 points
Lower quality studies: < 4 points

Supplementary table 2: Pooled estimates of the severity in the LT recipients with COVID-19 undergoing IS therapy.

IS Therapy	No. of studies	No. of severe patients	Total Patients (No.)	Prevalence of severity (%)	I^2 (%)	OR(CI)	p
Overall IS	6	93	435	22.4%	71%	0.217(0.123-0.325)	0.004
CNIs	5	42	231	21.1%	55.91%	0.211(0.079-0.343)	0.059
Steroids	5	21	79	24.7%	17.01%	0.247(0.133-0.362)	0.306
Anti-metabolites	5	31	137	26.3%	32.23%	0.263(0.114-0.411)	0.207
mTORi	1	5	37	13.5%	NA	NA	NA

Supplementary table 3: Pooled estimates of the mortality in the LT recipients with COVID-19 undergoing IS therapy.

IS Therapy	No. of studies	No. of Dead patients	Total Patients (No.)	Prevalence of mortality (%)	I^2 (%)	OR(CI)	p
Overall IS	6	86	435	19.5%	0%	0.183(0.158-0.232)	0.754
CNIs	6	32	196	15%	0%	0.150(0.100-0.199)	0.520
Steroids	6	15	86	17.2%	0%	0.172(0.095-0.249)	0.683
Anti-metabolites	6	16	120	12.1%	0%	0.121(0.065-0.178)	0.569
mTORi	3	0	13	8.3%	0%	0.083(-0.051 -0.217)	0.894

Supplementary table 4: Pooled estimates of the severity in the LT recipients with COVID-19 on the basis of comorbidities.

Comorbidity	No. of studies	No. of severe patients	Total Patients (No.)	Prevalence of severity (%)	I (%)^2	OR(CI)	p
Diabetes	5	26	110	26.4%	11.95 %	0.264(0.140-0.388)	0.337
Hypertension	4	40	132	37%	55.42 %	0.370(0.162-0.579)	0.081
Cardiopulmonary disorders	4	13	55	32.5%	57.47 %	0.325(0.083-0.566)	0.070
CKD	3	15	67	30.2%	63.06 %	0.302(0.071-0.533)	0.067
Age >60	4	32	162	20%	14%	0.200(0.109-219)	0.323
Obese	5	16	61	31.2%	39.52 %	0.312(0.116-0.508)	0.158
No Comorbidity	4	4	63	6%	0%	0.060(0.003-0.116)	0.542

Supplementary table 5: Pooled estimates of the mortality in the LT recipients with COVID-19 on the basis of comorbidities.

Comorbidity	No. of studies	No. of Dead patients	Total Patients (No.)	Prevalence of mortality (%)	I^2 (%)	OR(CI)	p
Diabetes	5	17	75	22.9%	0 %	0.229(0.136-0.323)	0.915
Hypertension	4	16	74	21.7%	0 %	0.217(0.124-0.311)	0.940
Cardiopulmonary disorders	4	10	37	27.9%	0 %	0.279(0.143-0.416)	0.444
Age >60	4	9	44	19.4%	0 %	0.194(0.078-0.309)	0.770
Obese	5	12	59	19.5%	0 %	0.195(0.097-0.293)	0.725
No Comorbidity	3	0	12	7.7%	0 %	0.077(-0.056 -0.209)	0.786
